# HO Endonuclease-Initiated Recombination in Yeast Meiosis Fails To Promote Homologous Centromere Pairing and Is Not Constrained To Utilize the Dmc1 Recombinase

**DOI:** 10.1534/g3.118.200641

**Published:** 2018-09-25

**Authors:** Lina Yisehak, Amy J. MacQueen

**Affiliations:** Department of Molecular Biology and Biochemistry, Wesleyan University, Middletown, CT

**Keywords:** chromosome pairing, HO endonuclease, meiosis, recombination, Spo11, synapsis

## Abstract

Crossover recombination during meiosis is accompanied by a dramatic chromosome reorganization. In *Saccharomyces cerevisiae*, the onset of meiotic recombination by the Spo11 transesterase leads to stable pairwise associations between previously unassociated homologous centromeres followed by the intimate alignment of homologous axes via synaptonemal complex (SC) assembly. However, the molecular relationship between recombination and global meiotic chromosome reorganization remains poorly understood. In budding yeast, one question is why SC assembly initiates earliest at centromere regions while the DNA double strand breaks (DSBs) that initiate recombination occur genome-wide. We targeted the site-specific HO endonuclease to various positions on *S. cerevisiae*’s longest chromosome in order to ask whether a meiotic DSB’s proximity to the centromere influences its capacity to promote homologous centromere pairing and SC assembly. We show that repair of an HO-mediated DSB does not promote homologous centromere pairing nor any extent of SC assembly in *spo11* meiotic nuclei, regardless of its proximity to the centromere. DSBs induced *en masse* by phleomycin exposure likewise do not promote homologous centromere pairing nor robust SC assembly. Interestingly, in contrast to Spo11, HO-initiated interhomolog recombination is not affected by loss of the meiotic kinase, Mek1, and is not constrained to use the meiosis-specific Dmc1 recombinase. These results strengthen the previously proposed idea that (at least some) Spo11 DSBs may be specialized in activating mechanisms that both 1) reinforce homologous chromosome alignment via homologous centromere pairing and SC assembly, and 2) establish Dmc1 as the primary strand exchange enzyme.

Successful ploidy reduction during meiosis requires that chromosomes efficiently search for, recognize, and establish a stable connection with their homologous partners, allowing them to orient and segregate properly to opposite poles of the meiosis I spindle apparatus (Page and Hawley 2003). Accordingly, a characteristic feature of meiotic prophase nuclei is a large-scale reorganization in which homologous chromosomes transition from an unpaired distribution to an intimate, lengthwise alignment, usually in the context of an elaborate proteinaceous structure, the synaptonemal complex (SC). However, the connection that homologous chromosomes (homologs) ultimately rely on for their proper segregation is more discrete: this crucial link is nearly always provided by a crossover recombination event. Thus, the central task of meiosis is to coordinate a dramatic change in the spatial distribution of chromosomes with the formation and repair of DNA double strand breaks (DSBs) in a manner that promotes crossing over (Pawlowski and Cande 2005; Hunter 2015; Zickler and Kleckner 2015). The molecular basis for how DNA repair and chromosome pairing mechanisms are coordinated during meiosis remains poorly understood.

Meiotic recombination is normally initiated via DSBs created by the conserved transesterase, Spo11, in conjunction with several accessory proteins (Lam and Keeney 2014). Each end of a Spo11-mediated DSB undergoes nucleolytic processing to generate 3′ single stranded DNA; these single-stranded termini associate with homologs of the *E. coli* RecA recombinase protein in order to assemble nucleoprotein filaments capable of catalyzing strand exchange with homologous duplex DNA (Brown and Bishop 2014; Lam and Keeney 2014; Hunter 2015). In budding yeast meiosis, a subset of nascent strand exchange events undergo dissolution after limited DNA synthesis, and DNA repair is completed for these events via a synthesis-dependent-strand-annealing (SDSA) mechanism to form a noncrossover (Allers and Lichten 2001a; Mcmahill *et al.* 2007). Another subset of strand exchange events become stable single end invasion intermediates, and then mature into double-Holliday junction (dHJ) containing joint molecule structures, after “capture” of the second end of the DSB (Schwacha and Kleckner 1994; Schwacha and Kleckner 1995; Allers and Lichten 2001b; Hunter and Kleckner 2001). In budding yeast, the interhomolog crossovers that are critical for proper chromosome segregation form predominantly by the resolution of dHJs (Allers and Lichten 2001a).

The diploid meiotic cell offers three homologous templates that could be targeted for strand exchange by either end of a DSB: One sister and two non-sister chromatids. In contrast to vegetative cells (Kadyk and Hartwell 1992 ; Bzymek *et al.* 2010) homologous recombination in meiotic cells preferentially utilizes a non-sister chromatid (the homolog) as a template for DNA repair (Schwacha and Kleckner 1994; Schwacha and Kleckner 1995; Hunter and Kleckner 2001; Hong *et al.* 2013). One meiosis-specific mechanism that promotes interhomolog *vs.* intersister DNA repair involves the specialized, concerted action of two RecA homologs: The strand exchange activity of a meiosis-specific RecA homolog, Dmc1 (Bishop *et al.* 1992) in conjunction with a supporting activity of the mitotic RecA protein, Rad51 (Game and Mortimer 1974; Game *et al.* 1980; Shinohara *et al.* 1992; Schwacha and Kleckner 1997; Shinohara *et al.* 1997a; Cloud *et al.* 2012). In the context of solely Dmc1 or Rad51, meiotic interhomolog recombination is dramatically diminished and residual DSB repair occurs primarily using the sister chromatid (Bishop *et al.* 1992; Shinohara *et al.* 1992; Shinohara *et al.* 1997a; Hong *et al.* 2013; Lao *et al.* 2013). During normal meiosis in budding yeast, Rad51’s strand exchange activity is diminished and the preferential use of a Dmc1 recombinase pathway may be ensured in part by the inhibition of the Rad54 motor protein (Shinohara *et al.* 1997b; Niu *et al.* 2005; Busygina *et al.* 2008; Niu *et al.* 2009) and the stabilization of an interaction between Rad51 and its inhibitor, Hed1 (Tsubouchi and Roeder 2006; Lao *et al.* 2013); both of these pathways rely on the activity of the meiosis-specific kinase, Mek1 (Xu *et al.* 1997; Hong *et al.* 2013; Callender *et al.* 2016; Hollingsworth 2016).

In budding yeast, meiotic recombination not only generates interhomolog crossovers but also promotes homologous chromosome synapsis - the assembly of SC between lengthwise-aligned chromosomes (Page and Hawley 2004; Cahoon and Hawley 2016). The SC has a conserved, tripartite structure in which rod-like transverse filament proteins assemble in perpendicular orientation to the long axis of the chromosome. Transverse filament proteins bridge chromosome axes (called lateral elements within assembled SC) and a distinct substructure, the central element, assembles at the midline of the SC. The transverse filament of the budding yeast SC is comprised of the Zip1 protein, which has an extensive central coiled-coil motif that is predicted to fold into a rod-shaped homodimer or tetramer (Sym *et al.* 1993; Sym and Roeder 1995; Dong and Roeder 2000). The interacting Ecm11 and Gmc2 proteins assemble the central element substructure of budding yeast SC (Humphryes *et al.* 2013; Voelkel-Meiman *et al.* 2013).

Synapsis initiates at multiple discrete points along the length of chromosomes, many of which are likely sites of interhomolog recombination (Chua and Roeder 1998; Agarwal and Roeder 2000; Henderson *et al.* 2004). Interestingly, however, the earliest SC assembly events in budding yeast meiotic cells occur predominantly from centromeres (Tsubouchi *et al.* 2008). Spo11-dependent SC assembly from centromeres raises the mechanistic question of how this class of synapsis events is coupled to meiotic recombination, given that centromeres are not thought to correspond to sites that undergo frequent interhomolog recombination in budding yeast meiosis (Lambie and Roeder 1986; Lambie and Roeder 1988; Blitzblau *et al.* 2007; Chen *et al.* 2008; Pan *et al.* 2011; Vincenten *et al.* 2015).

One explanation for initial SC assembly from centromeres in budding yeast may relate to the existence of an SC-independent “coupling” mechanism that can reinforce pair-wise interactions between homologous centromeres. The SC transverse filament protein, Zip1, mediates two-by-two associations between centromeres, regardless of homology and independent of Spo11 activity, at the onset of meiosis in budding yeast (Tsubouchi and Roeder 2005). Zip1 also mediates pair-wise associations between homologous centromeres in a Spo11-dependent manner during later meiotic prophase (Kemp *et al.* 2004; Falk *et al.* 2010; Newnham *et al.* 2010; Kurdzo *et al.* 2017). Zip1’s centromere pairing activity does not involve a conventional SC structure, as the SC central element protein Ecm11 is dispensable for both Spo11-independent and Spo11-dependent centromere pairing (Humphryes *et al.* 2013; Kurdzo *et al.* 2017). However, the local abundance of Zip1 at centromeres perhaps bestows these chromosomal regions with an increased capacity to assemble SC in response to *trans* acting signals from recombination sites.

How are meiotic DNA repair processes connected to specialized chromosome pairing and synapsis outcomes? Thorne and Byers (1993) showed that the capacity for X-ray induced DSBs to partially rescue the low spore viability of *spo11* meiotic cells depends on the meiosis-specific chromosomal protein, Hop1; this result indicates that recombination intermediates might ensure meiotic chromosome pairing and/or segregation outcomes at least in part by interfacing with meiotic factors that function at the chromosome axis. Consistent with this notion, a handful of meiosis-specific proteins that localize within the SC, including Zip1, Zip2, Zip3, Zip4, Spo16, and the MutSγ complex Msh4-Msh5, are critical for normal levels of dHJs and crossing over during meiosis (Sym *et al.* 1993; Agarwal and Roeder 2000; Novak *et al.* 2001; Tsubouchi *et al.* 2006; Shinohara *et al.* 2008). Furthermore, meiosis-specific chromosome axis proteins such as Red1, Hop1 and Rec8 have been associated with promoting interhomolog interactions (Shinohara *et al.* 1997a; Kim *et al.* 2010; Hong *et al.* 2013).

Further insight into how DNA repair machinery is connected to specialized chromosome pairing and synapsis outcomes in budding yeast is provided by elegant studies that compare recombination outcomes in meiotic *vs.* mitotic cells. Malkova *et al.* (2000) found that an HO-mediated DSB is four times more likely to repair as an interhomolog crossover in *spo11* meiotic cells than in mitotic cells; these results, similar to the aforementioned results of Thorne and Byers, suggest the possibility that Spo11-independent mechanisms ensure that any DSB (no matter the source) is processed in a manner that is “meiotic-like” in budding yeast meiotic cells. However, it was also noted that Spo11 activity has a *trans* effect on HO DSB repair: The presence of Spo11 correlated with a shorter interhomolog conversion tract length and an even higher likelihood of crossing over relative to when Spo11 is absent (Malkova *et al.* 2000). Independent studies using VDE, a site-specific endonuclease, provide additional evidence that Spo11 can increase the likelihood of interhomolog repair (Neale *et al.* 2002), reduce gene conversion tract length *in trans* (Neale *et al.* 2002), as well as influence the repair factors utilized (Medhi *et al.* 2016). Furthermore, when assessed, prior studies found no evidence that an HO-mediated or VDE-mediated DSB promotes SC assembly in *spo11* mutant meiocytes (Malkova *et al.* 2000; Neale *et al.* 2002), although partial SC assembly may have been missed because of the small size of the chromosomes sustaining the DSB. Taken together these findings support the idea that unique properties of meiosis-specific DSB machinery, in conjunction with recombination-independent features of the meiotic nucleus, ensure that recombination is accompanied by robust homolog engagement and crossing over in budding yeast. Our understanding of whether and how meiotic chromosome pairing processes are uniquely regulated by Spo11, however, remains incomplete.

In this study, we addressed the question of whether a meiotic DSB’s position relative to the centromere affects the homologous centromere pairing or synapsis outcome. We evaluated the genetic and cytological behavior of meiotic cells devoid of Spo11 but capable of HO endonuclease-mediated DSBs at several distinct positions along budding yeast’s longest chromosome (IV), including positions that Spo11 utilizes frequently. We asked whether HO-mediated DSBs positioned nearby or distal to centromere IV, or alternatively whether *en masse* phleomycin-induced DSBs, are capable of promoting homologous centromere pairing and/or any extent of SC assembly in *spo11* mutant meiotic nuclei. Our investigation revealed that HO-mediated and phleomycin-induced DSBs fail to promote homologous centromere pairing and even partial SC assembly, regardless of their proximity to the centromere. Our results reveal additional potential distinctions between the processing of HO- *vs.* Spo11-initiated recombination intermediates in meiotic cells, including a difference in overall reliance on the meiosis-specific Mek1 kinase and Dmc1 recombinase. Our findings add to mounting evidence that one or more specialized properties of at least a subset of Spo11 DSBs serve to couple a recombination-based homology recognition process with mechanisms that reinforce homolog pairing in yeast meiosis, and that one critical and unique feature of pro-synapsis Spo11 DSBs may be their engagement with the mechanism that establishes Dmc1 as the primary recombinase.

## Materials and Methods

### Strain Construction

Yeast strains used in this study are isogenic to BR1919-8B (Rockmill *et al.* 1995); Table S4 lists their genotypes. Strains were constructed using standard genetic and transformation methods. Primers are listed in Table S5.

To build a plasmid to integrate HO endonuclease driven by *SPO13* promoter sequences at the *LYS2* locus, sequences encompassing *LYS2*, including 420 bp upstream and 50 bp downstream, were amplified using primers AJM838 and AJM839, and inserted at the *Sma*I site of pRS315 to create pAM191. An *Xba*I site was engineered into the reverse primer AJM839 so that *Xba*I sites flank the HO cassette in pAM191. A step-wise PCR was used to generate *P_SPO13_-HO*. First, *SPO13* promoter sequence was amplified from genomic DNA using primers AJM763 and AJM764. AJM764 has sequence that overlaps with the beginning of HO. Second, HO endonuclease ORF sequence (ATG through 200 bp downstream of the STOP) were amplified from a plasmid carrying *P_GAL_- HO* (Jensen and Herskowitz 1984), using primers AJM765 and AJM766. Finally, the two overlapping DNA fragments were “stitched” in an amplification using primers AJM763 and AJM766. The *P_SPO13_* –*HO* fragment was inserted at the SnaBI site of pAM191 to create pAM200. Strains in which *P_SPO13_* –*HO* successfully integrated at the *LYS2* locus were identified first by using counter-selection against *LYS2* on alpha-aminoadipate plates and then by PCR.

**Figure 1:**
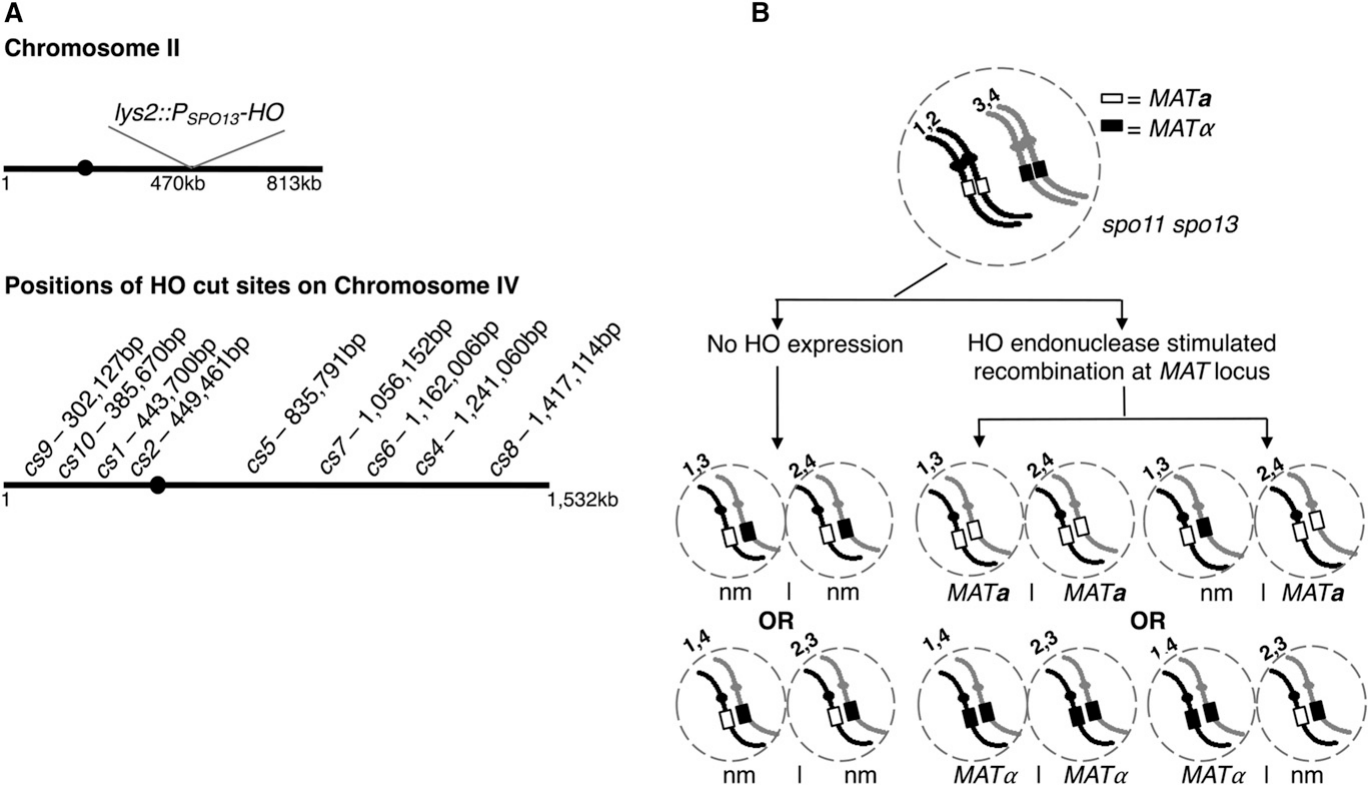
Creating strains in which HO endonuclease is the sole source of meiotic DSBs. (A) Illustration indicates the chromosomal positions of a meiosis-specific HO endonuclease gene cassette and various *HO* cut sites (*HO cs)*. *P_SPO13_ -HO* interrupts the *LYS2* locus on chromosome II. *HO cs* sequences together with the *natMX4* drug marker were targeted to the indicated chromosome IV coordinates. *CENIV* (solid circle) corresponds to coordinates 449,711- 449,821bp (*Saccharomyces* Genome Database). (B) Illustration depicts genotypic and phenotypic *MAT* locus outcomes of *spo11 spo13* meiotic nuclei with or without meiotic expression of HO endonuclease. Meiotic cells undergo a single equational division in *spo11 spo13* strains producing a dyad with diploid spores. In the absence of HO-mediated recombination, each dyad spore receives one copy of *MAT****a*** and one copy of *MATα*, resulting in two non-mating spores (nm). In the presence of HO endonuclease, interhomolog or intrachromosomal recombination at the *MAT* locus on any or all of the chromatids (1, 2, 3 or 4) can produce homozygous *MAT****a*** or *MATα* spores, which will be phenotypically a or *α* “maters”.

To create a template containing the *HO cut site* sequence (*HO cs*) linked to a genetic marker, a 100 bp *Hin*dIII fragment containing the *HO cs* sequence was excised from pAR134 (Ray *et al.* 1988) and inserted at the *Hin*dIII site of pAG25, which carries *natMX* (Goldstein and Mccusker 1999) to create pAM159. *HO cs* sequences were amplified from pAM159 using primers with sequence homology to various locations on chromosome IV (see Table S5). *HO cs* sequences were integrated at the chromosome IV coordinates indicated in Figure 1A; except for *cs1* and *cs2*, each of these chromosomal positions have been identified as regions of frequent cleavage by Spo11 (Blitzblau *et al.* 2007; Pan *et al.* 2011; Thacker *et al.* 2014; Markowitz *et al.* 2017). The chromosomal position of *cs2*, which is 250 nucleotides from *CENIV*, has been found to be enriched for the meiosis-specific cohesin subunit Rec8 but not for the meiosis-specific axis proteins Red1 and Hop1 (Panizza *et al.* 2011). For technical reasons, two consecutive PCRs were performed for creating DNA unique to each cut site location on chromosome IV. The first PCR was carried out using AJM760, a forward primer common to all integration site cassettes, in conjunction with a reverse primer carrying homology to both pAM159 and the specific integration site on chromosome IV. The second PCR was performed using the same reverse primer in conjunction with a site-specific forward primer carrying homology to the template and the specific chromosome IV integration site. The specific forward and reverse primers used for PCR2 are: *cs1* (AJM750 and AJM751), *cs2* (AJM752 and AJM753), *cs4* (AJM756 and AJM757), *cs5* (AJM975 and AJM976), *cs6* (AJM1137 and AJM1138), *cs7* (AJM1141 and AJM1142), *cs8* (AJM1145 and AJM1146), *cs9* (AJM1255 and AJM1256) and *cs10* (AJM1259 and AJM1260). The position of the HO *cs* in each strain was confirmed by sequence analysis.

To generate strains with genetic markers flanking HO *cs2*, the *LEU2* gene cassette was amplified using primers AJM1702 and AJM1698 and inserted at position 447 kb (2.8 kb left of *CEN4*). *THR1* was PCR amplified with AJM1241 and AJM1242 and inserted at 1,416 kb on chromosome IV. To generate strains with genetic markers flanking all other *HO cs* loci, *LEU2* was integrated 705 nucleotides to the right of *CEN4* (described below). *THR1* was inserted as described for strains carrying *HO cs2*.

To build strains for pairing analysis, a *lacO* array was integrated 705 bp to the right of *CEN4* by digesting pJBN156 (Bachant *et al.* 2002) with *Nhe*I, and a *tetO* array was integrated at coordinate 1,242 kb of chromosome IV by digesting pAM152 (kindly donated by Karen Voelkel-Meiman) with *Eco*R1. pAM152 was generated as follows: First, a 920 bp fragment encompassing the sequences at position 1,242 kb on chromosome IV was amplified from genomic DNA using primers AJM650 and AJM651, and cloned into the *Hin*dIII/*Sph*I site of p306tetO224 (Michaelis *et al.* 1997), which contains 224 tandem *tetO* (11.2 kb) sequence repeats in a pRS306 vector (Sikorski and Hieter 1989); this created pAM145. Next, the *THR1* gene was amplified using primers AJM666 and AJM667 and cloned between AatII and *Sac*I sites of pAM145, creating pAM152.

*spo11-Y135F*::*kanMX4* was integrated to replace the *SPO11* gene by digesting pJY20 (a kind gift of Dr. Scott Keeney, Memorial Sloan Kettering Cancer Center) with *Sal*I and *Sac*II. pJY20 contains *spo11-Y135F*::*kanMX4* inserted into the multicloning site of pRS316 (Sikorski and Hieter 1989). Dr. Douglas Bishop (University of Chicago) kindly provided a *rad51-II3A* strain, which was used to amplify the *rad51-II3A* allele sequence that we introduced into our strain background.

The 2μ-*RED1-HOP1*, pNH219 (Hollingsworth and Ponte 1997) were a kind gift of Dr. Nancy Hollingsworth. A 2μ-*REC8-MYC* (pAM356) plasmid was constructed using gap repair to replace the *HOP1* ORF in pDW72 with *REC8-MYC*, which was amplified from strain LY893 (Table S4) with primers AJM2164 and AJM2165 (Table S5). The viability of spores from a strain homozygous for the *REC8-MYC* sequence that is carried by LY893 is 97% (n= 52), similar to the wild-type value (95%, n = 52).

Sporulation efficiency and spore viability of strains utilized for analysis of HO-mediated recombination are listed in Table 3, Table S2 and Table S3.

### Assay for mitotic HO-mediated MAT recombination

A single culture of each diploid strain used for the genetic analysis of crossover recombination (LY407, LY208, LY555, LY324, LY322 and LY207), which express *P_SPO13_-HO* or no HO endonuclease (control) was grown overnight in liquid rich medium at 30°. Cells were then diluted and transferred to rich medium plates at a density of 100-200 colonies per plate. After 2 days of incubation, all colonies were assessed for mating type using a complementation-based assay in which only those cells that mate with a tester *MAT****a*** or *MATα* strain are capable of growth on minimal media. Zero colonies from strains without *P_SPO13_-HO* exhibited any capacity to mate with either the *MAT****a*** or *MATα* tester strain, as assessed by growth on minimal media (n = 421). A low frequency of plated colonies (0.7%, 0.7%, 1.5% and 1.2% in four independent tests involving >400 colonies) from strains carrying *P_SPO13_-HO* exhibited papilla of growth on minimal media after mixing with either the *MAT****a*** or *MATα* tester strain, indicating a low level of HO activity during vegetative growth.

### Southern Blots

Genomic DNA was prepared at 0, 12, 18 and 24 hr of sporulation from strains, LY491, LY456, LY492, LY459, LY481, LY457 and LY458, which are homozygous for a *rad51* null mutation and thus severely delayed in completing DNA repair using homologous recombination. After digestion with the appropriate restriction enzyme (*Eco*RV for strains carrying *HO cs1*, *cs5* or *cs7*, *Xho*I for strains carrying *HO cs2*, *Pvu*II and *Xho*I for strains carrying *HO cs6* and *Spe*I for strains carrying *HO cs4*), genomic DNA was separated on a 0.8% TAE-buffered agarose gel and transferred to Hybond-Nylon membrane (klapholz and esposito). A 500 bp probe homologous to *natMX4* sequences, prepared using a DIG High Prime DNA Labeling and Detection kit was hybridized to the membranes in order to detect genomic DNA fragments containing *HO cs* sequences that were either cut or intact. A Syngene G:Box was used to detect chemiluminescence of the hybridized probe; intensity profiles for each lane were generated using the Syngene Gene Tools program. The percentage of *HO cs* DNA that was cut was calculated by dividing the intensity of the cut *HO cs*–containing fragment by the sum of the cut and uncut fragments. The average of two experiments is presented for each strain.

### Western blots

Protein was precipitated from 5 ml meiotic cultures at 24 hr of sporulation by trichloroacetic acid precipitation as previously described (Voelkel-Meiman *et al.* 2016). Precipitated protein was dissolved in 2x Laemmli sample buffer (Laemmli 1970), supplemented with 30 mM DTT. Samples were heated for 10 min at 65°, then centrifuged at top speed before the concentration of soluble protein was determined using a NanoDrop (Thermo Fisher). 25-50 μg of protein was loaded on an 8% polyacrylamide/SDS gel and separated at 200 volts for one hour. Separated proteins were transferred to 0.2 μm nitrocellulose membranes (Amersham) after equilibrating the membrane and gel in Towbin buffer (Towbin *et al.* 1979) for 15 min. Protein transfer was performed at 60 volts for one hour at room temperature, using a stir bar and ice pack. Ponceau S (Sigma) was used to confirm protein transfer to the membrane. Mouse anti-MYC (clone 9E10, Invitrogen), mouse anti-Hop1 (gift from S. Roeder, Yale) and rat anti-Tubulin (Santa Cruz) were used at 1:2500, 1:1000 and 1:5000 dilutions, respectively. Primary antibody incubations were performed overnight at 4°. HRP-conjugated goat anti-mouse (Jackson ImmunoResearch) and donkey anti-rat (Santa Cruz) were applied at 1:5000 for 2 hr at room temperature. Amersham ECL Prime Western Blotting Detection Reagent was used to visualize bands. A Syngene “G”:box” was used to detect chemiluminescence and the Syngene Tools program was used to analyze the data. Normalization was achieved by dividing the intensity of anti-Hop1 signal by the intensity of anti-Tubulin signal in each lane. The fold change of Hop1 abundance in strains with Hop1 overexpression was calculated by dividing the normalized Hop1 intensity in the overexpression strains by the normalized Hop1 intensity in the corresponding control strains. The same approach was used to quantify Rec8-MYC overexpression. The average of 3 experiments is shown.

### Cytology

Preparations of surface-spread meiotic chromosomes, their immunostaining and imaging were performed as previously described (Voelkel-Meiman *et al.* 2016). In a subset of experiments (noted in text), phleomycin (Invivogen) was added to the sporulation medium at a final concentration of 0, 30, 100 and 120 µg/ml. Primary antibodies were applied for 16 hr at 4° in a humid chamber. The following primary antibodies were used at the indicated dilutions: chicken anti-GFP (1:100) (Abcam), rabbit anti-mCherry (1:100) (Abcam), mouse anti-Hop1 (1:100) (a gift from S. Roeder, Yale), rabbit anti-Rad51 (1:100) (Calbiochem), affinity purified rabbit anti-Zip1(1:100) (YenZym Antibodies; raised against a C-terminal fragment of Zip1 as described (Sym *et al.* 1993), mouse anti-MYC (1:100) (clone 9E10, Invitrogen) and a polyclonal antibody raised (ProSci Inc.) against a mixed population of partial Emc11 and Gmc2 proteins (partial proteins kindly provided by Dr. Owen Davies, New Castle University). Fluorophore-conjugated secondary antibodies (Jackson ImmunoResearch) were applied at a 1:200 dilution for 2 hr at room temperature. Following antibody staining, samples were mounted in Vectashield medium (Vector Laboratories) supplemented with 1 µg/ml 4,6-diamidino-2-phenylindole (DAPI).

Imaging was carried out using a Deltavision RT imaging system (Applied Precision/GE) adapted to an Olympus (IX71) microscope and processed using Softworx software (GE). The 3-dimensional distances between the centers of GFP and mCherry foci within each surface-spread nucleus were measured using the Softworx Measure Distance Tool. Distances less than or equal to 0.5 µm were considered paired.

### Data Availability

Strains and plasmids are available upon request. The authors affirm that all data necessary for confirming the conclusions of this article are represented fully within the article, tables, figures, supplemental figures and supplemental tables. Supplemental material available at Figshare: https://doi.org/10.25387/g3.7052072.

## Results

### Creating strains in which HO endonuclease is the sole initiator of meiotic recombination

We created strains in which HO endonuclease is the sole source of meiotic DSB activity by integrating a DNA cassette, *P_SPO13_-HO*, which contains the *HO* ORF downstream of promoter sequences for the meiosis-specific *SPO13* gene, as was done by a prior study to assess HO-mediated meiotic DSBs (Malkova *et al.* 1996b). We integrated *P_SPO13_-HO* at the *LYS2* locus in strains missing both endogenous *HO* activity and *SPO11* (Figure 1A).

A *spo13* mutation was used to facilitate analysis of interhomolog recombination in a *spo11* strain background. The *spo13* mutation allows the isolation of viable spore products from diploid cells that fail to initiate or complete recombination, because *spo11spo13* meiotic cells undergo a single equational division to produce two diploid (dyad) spores instead of four haploid spores (Klapholz and Esposito 1980). Genetic markers can be inspected in the diploid spores to determine if interhomolog recombination has occurred.

Robust HO endonuclease activity was detectable in *P_SPO13_-HO*-containing meiotic cells, as meiotic spore products frequently carried a chromosome III that had undergone interhomolog recombination at the *MAT* locus, which carries the sequence that HO endonuclease targets *(HO cs)* (Haber 2012). In the absence of meiotic recombination at *MAT*, a *spo11spo13* (*MAT****a****/MATα*) meiotic cell will produce two *MAT****a****/MATα* diploid spores (Figure 1B). Indeed, almost all of the dyad spores that result from control *spo11spo13* diploids (with no HO endonuclease expression) fail to mate with either *MAT****a*** or *MATα* cells, as predicted for the *MAT****a****/MATα* genotype (Table 1). By contrast, approximately 40% of dyads from strains carrying *P_SPO13_-HO* carry at least one diploid spore that is phenotypically either *MAT****a*** or *MATα* (Table1), reflecting meiotic interhomolog recombination at *MAT*. These data are similar to the findings of (Malkova *et al.* 1996b), where 29% of otherwise recombination-less (*rad50* mutant) meioses involving *P_SPO13_-HO* expression and a single cleavable *MAT* locus displayed interhomolog conversion at *MAT*; this prior study also determined that every HO-associated meiotic conversion event arose from interhomolog recombination, *vs.* intra-chromosomal recombination using one of the two silent mating type loci.

**Table 1:** HO-mediated meiotic recombination at the *MAT* locus. Chromosome segregation is predominantly equational during *spo11 spo13* meiosis, resulting in dyads that are diploid and transheterozygous for *MAT**a*** and *MATα* (the parental genotype) and are thus non-maters (nm). HO-mediated interhomolog recombination at *MAT* locus during *spo11 spo13* meiosis can result in mating-capable spores, homozygous for *MAT**a*** or *MATα* (Figure 1B). Shown is the percentage of two-spore-viable dyads that carry spores of a specific phenotype (bold), and inferred genotype (unbold), from the diploid strains (LY407, LY208, LY555, LY207, LY322 and LY324) listed. Companion spores within a dyad are separated by a vertical line. The total number of two-spore-viable dyads in the analysis is given in the final column. The proportion of dyads carrying two non-mater (nm) spores is similar between strains carrying different *HO cs* loci: LY555 = 66.3%, LY207 = 58.3%, LY322 = 53.3% and LY324 = 67.3% (n>500 for each of these strains).


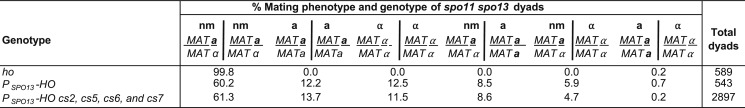

We observed similar levels of meiotic recombination at *MAT* in each of four *P_SPO13_-HO* strains containing chromosome IV-targeted *HO cs* loci (generated for experiments discussed below; Table 1). Diploid strains carrying *P_SPO13_-HO* display very little mating type switching during vegetative growth (see Materials and Methods), indicating that HO endonuclease activity in our *P_SPO13_-HO* strains is nearly completely meiosis-specific.

### HO forms DSBs at HO cs loci on chromosome IV during spo11 meiosis

*P_SPO13_-HO* strains were used to investigate whether the position of a meiotic DSB along the chromosome is a variable in assuring homologous centromere pairing and SC assembly. Toward this end, we integrated an *HO cs* at nine distinct locations on chromosome IV, including a site positioned 250 nucleotides to the left of *CENIV* (*HO cs2*; Figure 1A; Materials and Methods). Except for *cs1* and *cs2*, the chromosomal position of each of our *HO cs* integration sites is associated with a high probability of DSB formation by Spo11 during wild-type meiosis (Blitzblau *et al.* 2007; Pan *et al.* 2011). Neither *cs1* nor *cs2* are positioned at sites that have been found to be strongly enriched for meiosis-specific axis proteins but the position of *cs2* has been found to be strongly enriched for the meiosis-specific cohesin subunit, Rec8 (Panizza *et al.* 2011).

**Figure 2:**
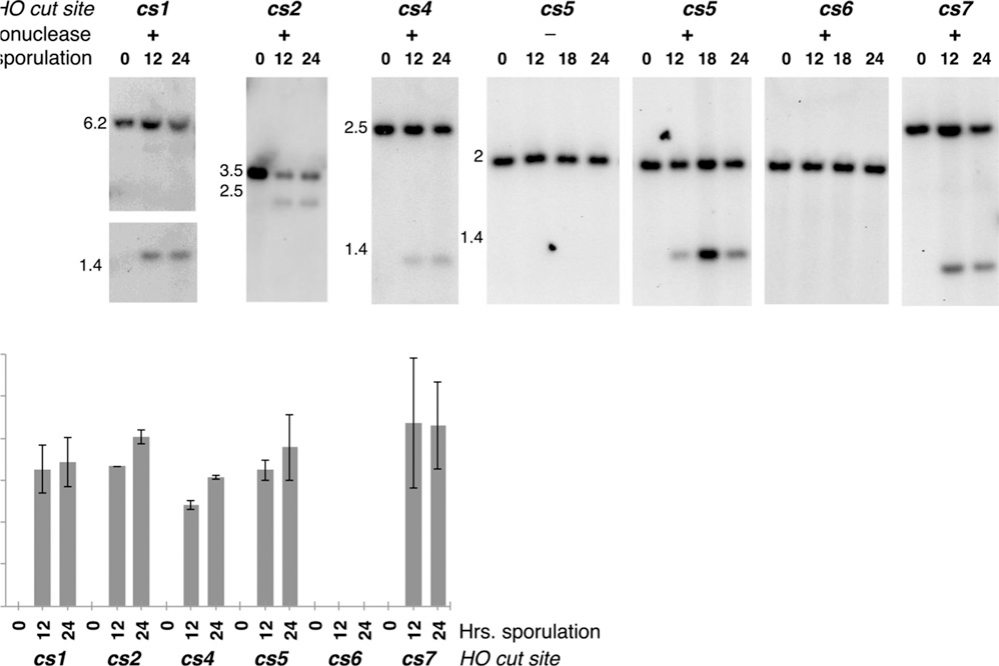
HO-mediated DSBs at *HO cs* sequences on chromosome IV in *spo11 rad51* meiosis. (A) Southern blot analysis shows DNA cleavage by HO endonuclease at *HO cs* sequences on chromosome IV in *spo11 spo13 rad51* diploid strains (LY491, LY456, LY492, LY481, LY459, LY457 and LY458; see Figure 1 for *HO cs* positions, Table S4 for strain genotypes). Samples were collected and processed at 0, 12, 18 and 24 hr after placement in sporulation medium. Genomic DNA was digested with restriction enzymes that target sites flanking each of the *HO cs* loci within a 10 kb region; DNA fragment sizes (kb) are displayed next to blots. Fragments were visualized using a probe that hybridizes to the *natMX4* sequence adjacent to each *HO cs*. In the absence of HO and before entry into meiosis in the presence of HO (0 hr of sporulation), the probe detects a single large fragment that corresponds to an intact fragment of DNA containing the *HO cs*. In the presence of HO endonuclease, a faster migrating (smaller) fragment is also seen for each of the cut sites beginning at 12hr, except for *cs6*. The absence of HO-mediated DSBs at *cs6* was verified using two different restriction enzymes (See Materials and Methods). (B) Bar graph shows the percent of DNA cut by HO endonuclease within *P_SPO13_-HO spo11 spo13 rad51* meiotic nuclei at the indicated time points. Values were calculated by dividing the intensity of the smaller fragment with the sum of the intensities of the smaller and larger fragment. Bars depict the range given by two experiments.

We directly evaluated HO-mediated DSB formation in six *spo11* null strains, each homozygous for a different chromosome IV *HO cs* (Figure 2). These strains were additionally homozygous for a *rad51* null mutation, in order to slow or abolish the completion of DNA repair and thereby allow us to evaluate the maximal level of HO-mediated DSB formation at these chromosomal sites during meiosis. Genomic DNA was isolated from *HO cs*-containing strains at 0, 12, 18 and 24 hr of sporulation; prior studies using this strain background indicate that most cells at the 24 hr time point have reached or progressed beyond late meiotic prophase (Voelkel-Meiman *et al.* 2012). Genomic DNA was digested using restriction enzymes that create a 2-7 kb DNA fragment encompassing the *HO cs*, and probed for *HO cs*-associated sequences using a 500 bp probe on a Southern blot.

In strains devoid of *P_SPO13_-HO*, or at the zero-hour time point in strains carrying *P_SPO13_-HO*, a single DNA fragment of predicted size was detected (Figure 2A). In strains carrying *P_SPO13_-HO* and chromosome IV-targeted *HO cs1*, *cs2*, *cs4*, *cs5* or *cs7*, a faster migrating fragment, corresponding to the product of an HO-mediated DSB, was also detected (Figure 2A). In these five strains, the percentage of DNA cut by HO endonuclease ranged from ∼20–60% (Figure 2B). These numbers suggest that HO endonuclease maximally cuts one or two out of the four available chromatids in a given meiotic nucleus. We note the possibility that this calculation underestimates the true number of HO-mediated DSBs, as some DSBs may be undetectable due to hyperresection of sequences flanking the DSB site (which has been noted to occur in *rad51* mutants (Shinohara *et al.* 1992). However, our probe did not detect faster migrating HO cleavage products, which would be expected observable intermediates in the case of substantial resection activity.

Unexpectedly, DSB formation at *HO cs6* was not detected in Southern blotting experiments with either of two different restriction enzymes (Figure 2A, B; Materials and Methods). HO endonuclease is active in the *cs6* strain, based on observed meiotic recombination at the *MAT* locus that was comparable to strains carrying the other *HO cs* loci (Table 1). The position of *cs6* on chromosome IV has been previously classified as a frequent target of Spo11 (Blitzblau *et al.* 2007; Pan *et al.* 2011); genetic background may affect the accessibility of *cs6* DNA to the HO endonuclease in our strain.

### HO promotes interhomolog recombination on chromosome IV in spo11 meiotic nuclei

**Figure 3:**
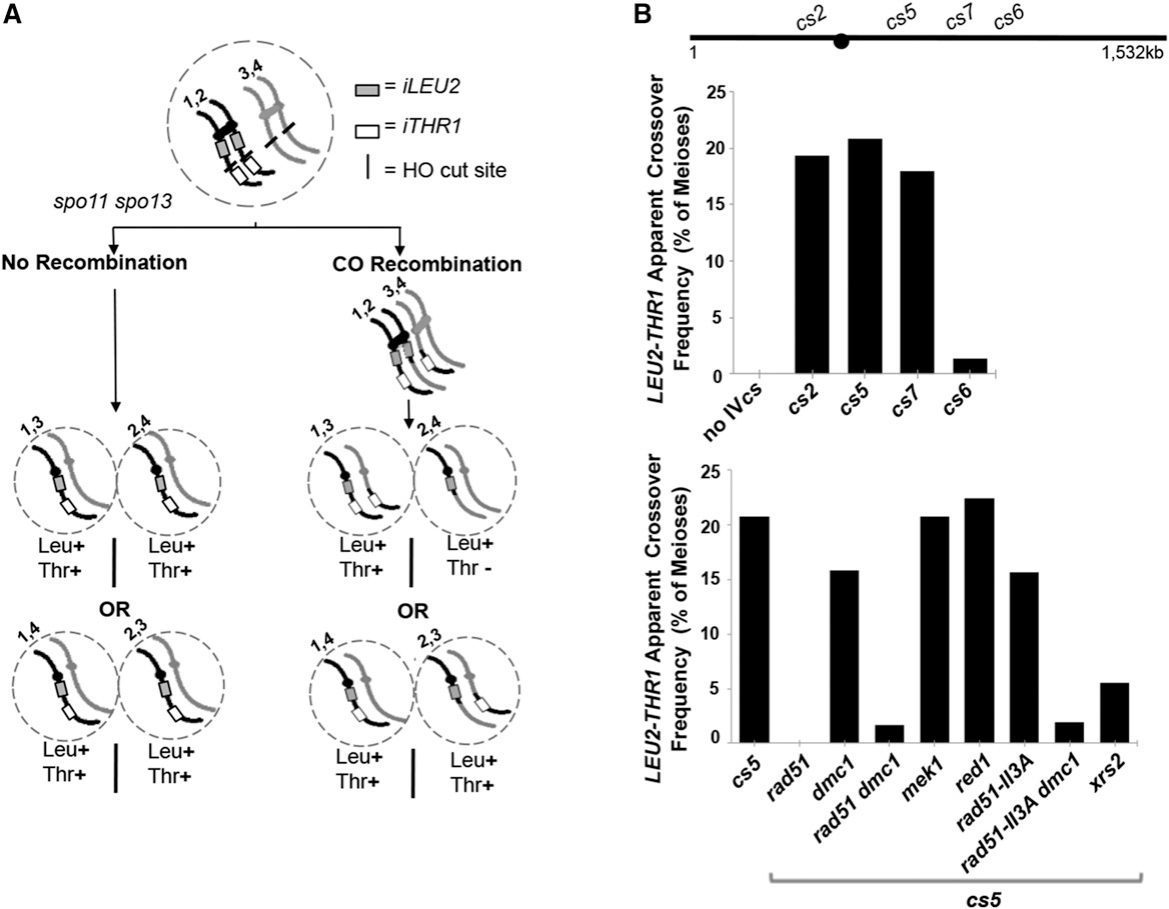
HO-mediated interhomolog recombination during meiosis. (A) Assay for interhomolog recombination at *HO cs* loci on chromosome IV in *spo11 spo13* diploids. *LEU2* is inserted at 450 kb (705 bp to the right of *CEN4*) and *THR1* is inserted at 1,416 kb on chromosome IV for all strains, except for *cs2* where *LEU2* is at 447 kb (2.8 kb to the left of *CEN4*). A single *HO cs* is integrated between *LEU2* and *THR1*. *spo11 spo13* diploid cells undergo a single equational division during meiosis, resulting in spores carrying a chromatid from each parental homolog. In the absence of HO, each spore receives one *LEU2 THR1* chromatid and one chromatid with neither marker (“No Recombination” column). In the presence of HO mediated DSBs, interhomolog reciprocal crossover recombination can result in a spore lacking the *THR1* marker on chromosome IV (accompanied by a sister spore with two *THR1* markers; upper right dyad). Because half of the crossover events will be invisible by this assay (lower right dyad), the percentage of apparent reciprocal crossovers were calculated by dividing twice the number of observed Thr- spores by the number of total 2-spore viable dyads (Table 2 and Table 4). (B) The upper bar graph plots the percentage of apparent crossing over calculated as described in (A) (n > 100 2-spore viable dyads assayed; Table 2) in various strains that carry no chromosome IV *HO cs* (LY208), or that carry distinct chromosome IV *HO cs* locations (left to right: LY208, LY555, LY207, LY324, LY322). The lower bar graph plots the percentage of apparent crossing over in mutant strains carrying *HO cs5* (left to right: LY207, LY459, LY290, LY393, LY904, LY939, LY935, LY957 and LY910). Vegetative cultures of control expressing no HO nor *HO cs* (LY407) as well as LY208, LY555, LY324, LY322 and LY207 were independently evaluated for the uniform presence of the *LEU2* and *THR1* marker by assessment of growth of >400 single colonies on selective media.

Meiotic DSBs preferentially engage the homolog over the sister chromatid for repair (Schwacha and Kleckner 1994; Schwacha and Kleckner 1997; Hong *et al.* 2013). To ask whether HO-mediated meiotic DSBs on chromosome IV engage the homologous chromosome for repair when Spo11 is absent, we assessed interhomolog crossing over between genetic markers that flank an *HO cs* (Figure 3A). The *LEU2* gene was introduced near to the centromere on one copy of chromosome IV, either 705 bases to the right of *CEN4* in *spo11spo13* strains homozygous for *HO cs5*, *cs6* or *cs7* or 2.8 kilobases to left of *CEN4* in analogous strains homozygous for *HO cs*2. In all strains, the *THR1* gene was inserted at coordinate 1,416,000 on the right arm of the chromosome IV carrying *LEU2+*. The *LEU2* and *THR1* genetic markers thus allowed us to measure the frequency of HO-mediated crossing over.

In the absence of HO, *spo11spo13* dyads heterozygous for *LEU2* near the centromere and *THR1* on the arm of chromosome IV would be expected to undergo equational division to give diploid dyads containing spores that are each heterozygous for the *LEU2* and *THR1* insertions (Figure 3A, “No Recombination” outcome). An HO-mediated, interhomolog crossover recombination event at an *HO cs* to the right of the centromere and between the *LEU2* and *THR1* markers, however, can result in a situation where both chromosomes IV carry one chromatid that is devoid of the *THR1* insertion (Figure 3A, “Recombination” outcome). Half of the diploid dyads arising from meioses involving such recombinant chromosomes IV will carry a spore that is phenotypically Leu+ Thr-. Figure 3A illustrates the phenotypes of each spore in such recombinant dyads. Note that a reciprocal crossover at *cs2*, which is positioned to the left of *CENIV*, will result in “recombinant” dyads that contain a Leu-, Thr+ spore.

We measured percent of meioses with an apparent reciprocal crossover event by dividing twice the number of “recombinant” dyads (dyads containing a Leu+, Thr- spore for non-*cs2* strains and containing a Leu-, Thr+ spore for the *cs2* strain) by the total number of dyads analyzed (Figure 3B, Table 2). Based on this calculation, HO-mediated, interhomolog crossover recombination occurred in ∼21% of meioses for *HO cs5*, 18% of meioses for *HO cs7*, and 19% of meioses for *HO cs2* (closest to *CENIV*) (Table 2, Figure 3B). Consistent with the absence of detectable DSBs at *HO cs6*, interhomolog crossover recombination could be detected at this cut site in only 1.3% of meioses (Table 2, Figure 3B).

**Table 2:** HO-stimulated meiotic recombination in strains with *HO cs* loci on Chromosome IV. The number of dyads with particular spore phenotypes was used to calculate apparent interhomolog crossover recombination in *spo11 spo13* meiotic cells with HO-mediated DSB formation at a chromosome IV *HO cs* location (See Figure 3A). *LEU2* is at chromosome IV coordinate 450,000 bp, to the immediate right of *CEN4*,** except for the *cs2* strain where *LEU2* is positioned at 447,000 bp (to the left of *CENIV)*; *THR1* is positioned at chromosome IV coordinate 1,416,000. The percentage of apparent crossing over in all strains except LY555 (*cs2*) was calculated by first doubling the number of dyads carrying a single Leu+, Thr− spore along with a Leu+, Thr+ spore (the first phenotype column), in order to account for the fraction of events not observed, and then by dividing the product by the total number of two-spore-viable dyads (n). The percentage of apparent crossing over in LY555 was calculated by doubling the number of dyads carrying a single Leu−, Thr+ spore along with a Leu+, Thr+ spore (the sixth phenotype column) and dividing this product by the total number of two-spore-viable dyads. The minor population of dyads from LY555 that carry one Leu+, Thr+ spore and one Leu+, Thr− spore could be explained by a reductional chromosome segregation event, may be associated with recombination nearby to the centromere (Simchen and Hugerat 1993). The minor population of dyad spores with loss of both *THR1* markers in some strains is possibly due to two interhomolog recombination events (involving all 4 chromatids). The minor class dyads were not included in the recombination calculation. A Fisher's Exact test was used to determine whether values differ significantly from the LY207 value. 400 or more colonies from vegetative cultures of LY208, LY555, LY324, LY322 and LY207 were independently evaluated for the uniform presence of the *LEU2* and *THR1* markers among all cells in the population by plating on selective media. All colonies evaluated were found to be Leu+, Thr+.

Genotype (Strain)	Phenotype of *spo11 spo13* dyads									n	2x observed apparent CO events	% apparent CO	*P*
	Leu+ | Leu+			Leu+ | Leu−			Leu− | Leu−						
	Thr+ | Thr− Thr− | Thr− Thr+ | Thr+			Thr+ | Thr− Thr− | Thr− Thr+ | Thr+			Thr+ | Thr− Thr− | Thr− Thr+ | Thr+						
*no cs* (LY208)	0	0	543	0	0	0	0	0	0	543	0	0.0	**-**
*cs2* (LY555)	13	1	481	3	0	54	1	0	5	558	108	19.4	**-**
*cs7* (LY324)	46	0	464	0	0	0	0	0	0	510	92	18.0	**-**
*cs6* (LY322)	4	1	601	0	0	0	0	0	0	606	8	1.3	**-**
*cs5* (LY207)	162	10	1384	0	0	0	0	0	0	1556	324	20.8	**-**
*cs5 rad51* (LY459	0	0	104	0	0	0	0	0	1	105	0	0.0	<0.0001
*cs5 rad51 dmc1* (LY393)	1	0	138	0	0	0	0	0	0	139	2	1.4	<0.0001
*cs5 dmc1* (LY290)	32	1	372	0	0	0	0	0	0	405	64	15.8	0.0250
*cs5 mek1* (LY904)	29	1	247	0	0	2	0	0	0	279	58	20.8	1.0000
*cs5 red1* (LY939)	36	0	289	0	0	0	0	0	0	322	72	22.4	0.5485
*cs5 rad51-II3A* (LY935)	14	0	164	0	0	0	0	0	0	178	28	15.7	0.1161
*cs5 rad51-II3A dmc1* (LY957)	1	0	103	0	0	0	0	0	0	104	2	1.9	0.0121
*cs5 xrs2* (LY910)	5	0	176	0	0	0	0	0	0	181	10	5.5	0.0001

Table 2 and Figure 3 refer to *LEU2-THR1* recombinants as “apparent” crossovers, because the Leu+, Thr- spores that we have presumed to be due to interhomolog crossover recombination could instead arise from an interhomolog recombination event that is nonreciprocal, in which sequences encompassing *THR1* are converted without an accompanying crossover. In the case of an interhomolog conversion without a reciprocal crossover, the Leu+, Thr+ sister spore in the recombinant dyad is expected to carry one chromatid devoid of the *THR1* insert. Whereas in the case of a reciprocal crossover, both chromatids in the Leu+, Thr+ spore within a recombinant dyad are expected to contain the *THR1* insert. We used PCR to detect the presence or absence of the *THR1* insert in Thr+ spores of 44 recombinant dyads for *cs5*-carrying strains, and to detect the presence or absence of the *LEU2* insert in Leu+ spores of 54 recombinant dyads from *cs2*-carrying strains. We found that the 71% (31 out of 44) of Thr+ spores from *cs5* recombinant dyads carried only *THR1* insert-carrying chromosomes IV, indicating that 71% of recombinant dyads in this strain result from a reciprocal crossover event. ∼30% of Thr+ spores were heterozygous for *THR1* insert-carrying chromosome IV, indicating that the recombinant spore in these dyads likely resulted from a nonreciprocal interhomolog recombination event instead of a reciprocal crossover. In an analogous analysis for *cs2*, we found that both chromosomes IV carried the *LEU2* insert in only 52% (28 out of 54) of Leu+ spores from *cs2* recombinant dyads. Thus, about half of the recombinant dyads from the *cs2* strain result from a reciprocal crossover event, while the remainder apparently derive from a nonreciprocal recombination event.

The higher fraction of nonreciprocal interhomolog recombination events in *cs2* *vs.* *cs5* strains may be due to the proximity of the *LEU2* marker to the DSB site: the *LEU2* marker is approximately 2.5 kilobases from *cs2*, whereas the *THR1* insert is ∼580 kilobases from *cs5*. On the other hand, it is remarkable that ∼30% of interhomolog recombination events in *cs5* strains involve conversion of sequences ∼580 kilobases from the DSB site. Such remarkably long conversion tracts have been previously associated with HO-mediated DSB repair in *spo11* meiotic nuclei (Malkova *et al.* 1996b; Malkova *et al.* 2000) and have been proposed to arise through a Break-Induced Replication (Kellis *et al.* 2004) process whereby a strand invasion intermediate is extended by replication through the end of the chromosome instead of undergoing second-end capture (Malkova *et al.* 1996a; Kraus *et al.* 2001; Malkova and Ira 2013).

In sum, as was reported in (Malkova *et al.* 2000) for the endogenous *HO* cs on chromosome III, our data suggest that HO-mediated meiotic DSBs on chromosome IV can engage a non-sister chromatid for their repair. Furthermore, our data indicate that a non-sister chromatid is utilized for HO-mediated meiotic DSB repair in at least 20% of meioses, and that a substantial fraction (∼30%) of HO-mediated interhomolog events involve extremely long conversion tracts.

### Dmc1 is dispensable for HO-mediated interhomolog recombination in meiotic cells

We next asked whether HO-mediated interhomolog recombination events in *spo11* meiosis rely on meiosis-specific strand exchange machinery. In *rad51* single or *rad51dmc1* double mutants, the viability of diploid dyad spores is dramatically decreased, from 74% in the *spo11spo13* control meiocytes expressing HO, to 38% in the analogous strain missing Rad51, and to 34% in the combined absence of Rad51 and Dmc1 (Table 3). Genetic removal of HO endonuclease from the *rad51* single or *rad51dmc1* double mutant strain restored dyad spore viability to 76% and 77%, respectively (Table 3), indicating that the spore lethality observed in our *HO*-expressing, *rad51* strains is due to a failure to repair HO-mediated DSBs.

**Table 3:** Meiotic HO-mediated DSBs lead to spore death in the absence of Rad51 activity or Mre11. The percentage of total dyads (n) from *spo11 spo13* diploids homozygous for various mutant alleles carrying two viable spores (2-sv), one viable spore (1-sv) and 0 viable spores (0-sv) is shown. The percentage of the total number of spores (nx2) that are viable is given for each strain in the final column (% Spore viability). Asterisks indicate a significant difference between spore viabilities for indicated strains, as determined by a Fisher's Exact test (*P* ≤ 0.01). Note that LY481 and LY500 were created by replacing *lys2::P*_*SPO13*_*-HO* in LY459, and LY393, respectively, with *LYS2* DNA sequence.

Genotype	(Strain)	dyads dissected	% Distribution of dyads types			% Spore viability
			2-sv	1-sv	0-sv	
*spo11 spo13*	(LY407)	728	81	16	3	89
LY407 *lys2*::*P_SPO13_-HO*	(LY208)	624	87	10	3	92
LY407 *lys2*::*P_SPO13_-HO cs5*	(LY207)	2028	60	27	12	74
LY407 *lys2*::*P_SPO13_-HO cs5 rad51*	(LY459)	520	20	35	45	38
LY407 *LYS2 cs5 rad51*	(LY481)	416	63	27	11	76
LY407 *lys2*::*P_SPO13_-HO cs5 rad51 dmc1*	(LY393)	937	15	39	46	34
LY407 *LYS2 cs5 rad51 dmc1*	(LY500)	936	62	29	9	77
LY407 *lys2*::*P_SPO13_-HO cs5 dmc1*	(LY290)	650	62	26	12	75
LY407 *lys2*::*P_SPO13_-HO cs5 mek1*	(LY904)	520	59	24	17	71
LY407 *mek1*	(LY907)	104	77	17	6	86
LY407 *lys2*::*P_SPO13_-HO cs5 red1*	(LY939)	572	56	26	18	69
LY407 *lys2*::*P_SPO13_-HO cs5 rad51-II3A*	(LY935)	520	34	36	29	52
LY935 *dmc1*	(LY957)	520	107	178	235	38
LY407 *lys2*::*P_SPO13_-HO cs5 mre11*	(LY916)	208	6	29	65	21
LY407 *LYS2 mre11*	(LY919)	104	29	42	29	50
LY407 *lys2*::*P_SPO13_-HO cs5 xrs2*	(LY910)	520	35	40	26	55
LY407 *LYS2 xrs2*	(LY913)	104	50	31	19	65

We also found that HO-mediated interhomolog crossovers at *cs5* are nearly abolished in the absence of Rad51. Apparent crossovers between the *LEU2* and *THR1* markers flanking *HO cs5* decreased from 20.8% observed among two-spore viable dyads from *spo11spo13* control meiocytes expressing HO, to 0% and 1.4% observed among two-spore viable dyads from *spo11spo13rad51* and *spo11spo13rad51dmc1* strains expressing HO, respectively (Figure 3B, Table 2). HO-mediated meiotic interhomolog conversion events at the *MAT* locus also disappeared in *spo11spo13rad51* meiotic cells expressing HO: 99.0% and 96.4% of two-spore viable dyads from *spo11spo13rad51* and *spo11spo13rad51dmc1* strains, respectively, exhibited two non-mating spores (n = 105; Table S1A), similar to the proportion of non-mating spores from *spo11spo13* strains that lack meiotic HO expression altogether (Table 1). Furthermore, only 2 out of 181 (1.1%; Table S1B) one-spore viable dyads from *P_SPO13_-HOspo11spo13rad51* meiotic cells and 5 out of 363 (1.4%; Table S1B) one-spore viable dyads from *P_SPO13_-HOspo11spo13rad51dmc1* meiotic cells exhibited the capacity to mate; these few *MAT* homozygotes may have arisen as a consequence of meiotic recombination or chromosome loss. Similarly, only 4 out of 181 (2.2%) and 1 out of 363 (0.3%) one spore viable dyads from *P_SPO13_-HOspo11spo13rad51* strains and *P_SPO13_-HOspo11spo13rad51dmc1* strains, respectively, exhibited a non-parental configuration of the *LEU2* and *THR1* markers that flank *cs5* on chromosome IV.

The low spore viability and absence of interhomolog recombination when Rad51 is missing from HO-expressing, *spo11spo13* meiocytes indicate that Rad51 is essential for robust repair of HO-mediated DSBs during meiosis, which aligns with the expectation that strand exchange is a requirement for meiotic recombination. Most strikingly, in contrast to Spo11-mediated meiotic DSBs, which are capable of utilizing a Dmc1-only pathway for repair by homologous recombination when Rad51 is absent (Bishop *et al.* 1992; Shinohara *et al.* 1997a), our data reveal that HO-mediated meiotic DSBs are incapable of undergoing homologous recombination using a Dmc1-only pathway.

In contrast to the Rad51-deficient context, no decrease in spore viability is associated with HO DSB activity at *cs5* in the absence of Dmc1 (75% spore viability in *spo11spo13dmc1* *vs.* 74% in *spo11spo13DMC1* strains respectively; Table 3) and apparent crossovers at *HO cs5* are only slightly decreased in *spo11spo13* meiotic cells missing Dmc1 (15.8% in *dmc1* *vs.* 20.8% in *DMC1* strains; Figure 3; Table 2). Furthermore, HO-mediated interhomolog recombination in *spo11spo13* meiotic cells appears unaffected by the individual removal of two factors that normally promote the use of the Dmc1 recombinase (Schwacha and Kleckner 1997; Callender *et al.* 2016): HO-mediated apparent interhomolog crossover levels are unchanged in *spo11spo13* meiotic cells missing the meiosis-specific kinase, Mek1 (20.8% in both *spo11spo13* control and *spo11spo13mek1* meiotic cells; Figure 3, Table 2), and in the absence of the meiosis-specific chromosomal protein, Red1 (22.4% in *spo11spo13red1* strains; Figure 3, Table 2).

Thus, in contrast to wild-type meiosis, where removal of factors that promote Dmc1 utilization causes a severe reduction in interhomolog crossovers (Rockmill and Roeder 1991; Bishop *et al.* 1992; Rockmill *et al.* 1995; Shinohara *et al.* 1997a; Bishop *et al.* 1999; Tsubouchi and Roeder 2003), HO-initiated interhomolog DSB repair in our experimental (*spo11*) strain is not dramatically affected by the loss of Mek1, Red1, or the Dmc1 recombinase, apparently because these HO-mediated DSBs can easily access a “Rad51-only” recombinase pathway.

### Can HO-mediated interhomolog recombination in meiotic cells utilize Dmc1 when Rad51 recombinase activity is absent?

The data presented above establish a requirement for Rad51 and the lack of a requirement for Dmc1 in the interhomolog repair of HO-mediated DSBs in meiosis. We used a Rad51 separation-of-function genetic context to determine if the Dmc1 recombinase functions redundantly with Rad51 recombinase activity, or whether Dmc1 is completely unavailable to the repair of HO-mediated meiotic DSBs in *spo11* mutants.

We analyzed HO-mediated meiotic DSB outcomes in the context of the *rad51-II3A* separation-of-function allele (a kind gift of D. Bishop; Cloud *et al.* 2012). *rad51-II3A* encodes a protein that lacks strand exchange activity and thus supports a Dmc1-Rad51 joint recombinase pathway but not a Rad51-only pathway. We observed robust HO-mediated interhomolog recombination in *spo11spo13* meiotic cells homozygous for *rad51-II3A* (15.7%; Figure 3, Table 2). Furthermore, we observed a strong diminishment in apparent interhomolog crossovers when Dmc1 is removed from the *rad51-II3A* strain, from 15.7% in *rad51-II3A* to 2% in *rad51-II3A dmc1* (Figure 3, Table 2), indicating that the interhomolog recombination measured in *rad51-II3A* mutants is primarily due to Dmc1 recombinase activity.

Altogether these data indicate that HO-initiated, interhomolog recombination intermediates may primarily engage the Rad51 recombinase, but are capable of utilizing Dmc1 as a recombinase so long as Rad51 is also present.

*spo11spo13rad51-II3A* meiotic cells that express *HO* exhibit diminished spore viability relative to control strains (52% *vs.* 74% in the control; Table 3). This observation indicates that Dmc1 is only partially capable of rescuing genome-wide HO-mediated meiotic DSB repair when Rad51 recombinase activity is diminished. Consistent with this interpretation, we observe evidence of Dmc1-mediated recombinase activity even in meiocytes that presumably also sustained lethal unrepaired HO DSBs due to the absence of Rad51 activity: ∼29% (n = 189) of one-spore-viable dyads from *rad51-II3A spo11spo13* strains expressing *P_SPO13_-HO* contain a spore that is homozygous at the *MAT* locus (presumably due to interhomolog recombination at *MAT*), in contrast to *rad51* and *rad51dmc1spo11spo13* strains expressing *P_SPO13_-HO* where only ∼1% of one-spore viable dyads carry a spore that is homozygous at *MAT* (Table S1B).

### HO-mediated DSB repair in meiotic cells is influenced by Mre11 and Xrs2

The Mre11 nuclease, Rad50 ATPase, and FHA-containing Xrs2 protein can form a complex (“MRX”) that functions in DSB repair in mitotic cells, and has been implicated in both DSB formation and repair during meiosis (Borde 2007; Gobbini *et al.* 2016). We investigated the role of this complex in the repair of HO-mediated, meiotic DSBs in *spo11* mutants by examining interhomolog recombination in *spo11spo13mre11* and *spo11spo13xrs2* meiotic cells that carry *P_SPO13_-HO* and the *HO cs5*. We found that, even in the absence of *HO* expression, the *mre11* and *xrs2* mutations alone confer a substantial decrease in spore viability to *spo11spo13* strains (50% and 65%, respectively, *vs.* 89% for the *spo11spo13* control; Table 3). This data indicates that Mre11 and Xrs2 activities are critical for maintaining genomic integrity during a *spo11spo13* meiotic cell cycle even in the absence of meiotic DSBs, and is consistent with a prior finding that diminished Mre11 activity reduces the spore viability of a *spo13* strain (Ajimura *et al.* 1993). In strains carrying *P_SPO13_-HO*, we observed that the *mre11* mutation confers a further reduction in spore viability (from 50% in the absence of *P_SPO13_-HO* to 21% in the presence of *P_SPO13_-HO*; Table 3), indicating that Mre11 is also important for the repair of HO-mediated meiotic DSBs. Zero out of thirteen two-spore viable dyads and only 3 out of 60 (5%) one-spore-viable dyads produced by the *spo11spo13mre11* mutant exhibited a Leu+ Thr- marker configuration, but this dataset is too small to conclude that HO-mediated interhomolog and/or crossover repair is dependent on Mre11 in *spo11spo13* meiotic cells. The strikingly low spore viability might lead one to conclude that HO-mediated meiotic DSBs uniformly fail to repair in *spo11spo13mre11* meiocytes expressing *P_SPO13_-HO*. However, this may not be the case: 46% of two spore viable dyads and 45% of one spore viable dyads from *spo11spo13mre11* mutants were found to exhibit at least one mating capable spore (Table S1). While diploid spores homozygous for *MAT* can arise from a *spo11spo13* meiosis as a consequence of chromosome loss, the near absence of such *MAT* homozygotes among two-spore-viable and one-spore-viable dyads from *spo11spo13rad51* strains suggests that chromosome loss rarely occurs in these HO-expressing meiocytes and thus at least some HO-mediated DSBs in *spo11spo13mre11* meiotic cells are likely repaired via interhomolog recombination. Thus, we conclude that Mre11 is not uniformly required for the interhomolog repair of HO-mediated DSBs during meiosis, but that a subset of HO-mediated meiotic DSBs cause inviable meiotic products when Mre11 is absent.

Similar to Mre11, Xrs2 is not required for the interhomolog repair of HO-mediated meiotic DSBs, as is evident by the robust HO-mediated interhomolog recombination previously observed at *MAT* in *SPO11spo13xrs2* strains (Malkova *et al.* 1996b), Indeed, similar to *spo11spo13* control strains that express *P_SPO13_-HO* (Table 1), we found that sporulated *P_SPO13_-HOspo11spo13xrs2* strains give rise to a large fraction (31.5%; Table S1A) of two spore viable dyads in which one or both spores is mating-capable, indicative of robust meiotic interhomolog recombination at the *MAT* locus. In contrast to *spo11spo13mre11* strains, however, meiotic expression of *HO* in *spo11spo13xrs2* strains is associated with only a slight reduction in spore viability (from 65% in the absence of *HO*, to 55% in the presence of *HO*; Table 3), indicating that Xrs2 is less critical than Mre11 for the repair of that putative subset of HO-mediated DSBs that, left unrepaired, cause the low spore viability observed for *spo11spo13mre11* strains.

Interestingly, the level of HO-mediated, apparent meiotic crossovers at *HO cs5* is also significantly reduced when Xrs2 is absent, from 20.8% in the *spo11spo13* control to 5.5% in *spo11spo13xrs2* meiotic cells (*P* = 0.0001; Figure 3, Table 2). Thus, while Xrs2 (and Mre11) are not essential *per se* for the interhomolog repair of HO-mediated meiotic DSBs, the strong diminishment in apparent meiotic crossover events in *spo11spo13xrs2* strains may reflect a capacity of Xrs2 to drive HO-initiated interhomolog repair intermediates toward a crossover outcome.

### Does HO-mediated, meiotic DSB repair in the absence of Spo11 rely on canonical meiotic recombination proteins that act downstream of strand exchange?

**Figure 4:**
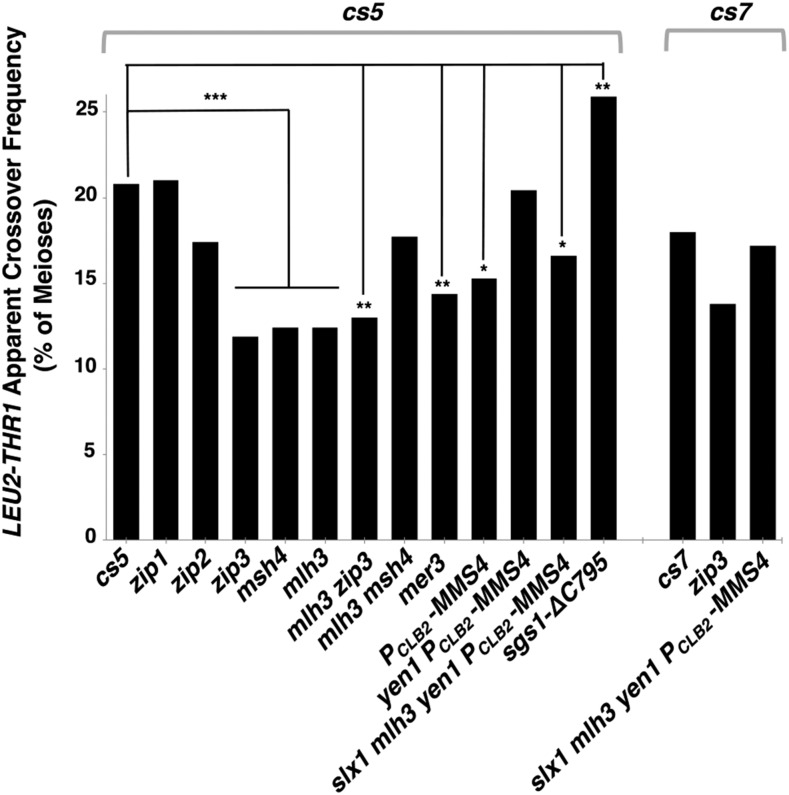
HO-mediated recombination in the absence of Spo11 does not rely heavily on canonical meiotic recombination factors. Bar graph shows the frequency of apparent crossovers in *spo11 spo13* strains carrying *P_SPO13_-HO*, an *HO cs* on chromosome IV, and mutant alleles of various recombination factors. Calculations were performed as described in Figure 3 (n > 250; precise values and strain names are reported in Table 4). Left part of graph shows data for strains carrying *HO cs5*, and the right 3 strains carry *HO cs7*. Significant deviations, relative to the wild-type value, were determined using Fisher’s Exact test (*P-value ≤ 0.05, **P-value ≤ 0.01, ***P-value ≤ 0.001).

We asked whether the Spo11-independent repair of HO-mediated DSBs utilize meiosis-specific recombination proteins that act downstream of strand exchange. Removal of the SC associated crossover-promoting factors Zip1, Zip2, Zip4, Mer3, Zip3, Msh4-Msh5 or Mlh3 during otherwise wild-type meiosis leads to a ∼50–70% reduction in crossing over in otherwise wild-type meiotic cells (Sym *et al.* 1993; Ross-Macdonald and Roeder 1994; Hollingsworth *et al.* 1995; Hunter and Borts 1997; Agarwal and Roeder 2000; Novak *et al.* 2001; Börner *et al.* 2004; Tsubouchi and Roeder 2006; Voelkel-Meiman *et al.* 2016). Of these factors, Zip1, Zip2 and Zip4 are known to be absolutely essential for SC assembly, while at least some assembled SC has been observed in mutants missing Zip3 or Msh4-5 complexes (Agarwal and Roeder 2000; Novak *et al.* 2001). We found that removal of Zip1 does not reduce the level of HO-mediated interhomolog recombination observed at *HO cs5* in *spo11spo13* meiotic cells (21% in *zip1* *vs.* 20.8% in the control), and removal of Zip2 conferred only a mild (∼16%; *P* = 0.13) reduction in apparent interhomolog crossovers observed at *HO cs5* (Figure 4, Table 4). However, removal of Zip3, Msh4, Mlh3, or Mer3 resulted in a significant reduction of HO-mediated, apparent interhomolog crossovers in *spo11spo13* meioses, by approximately 30–43% (*P* < 0.0001 for *zip3*, *msh4*, *mlh3*; *P* = 0.0095 for *mer3*; Figure 4, Table 4), suggesting that these meiosis-specific factors do influence the repair of a subset of HO-mediated recombination events. Taken together, these results suggest that HO meiotic recombination intermediates may be accessible to a subset of SC-associated recombination factors but not those that are absolutely critical for SC assembly.

**Table 4:** HO-mediated recombination in the absence of canonical meiotic recombination factors. The number of dyads with particular spore phenotypes was used to calculate apparent meiotic interhomolog crossover recombination in *spo11 spo13* strains carrying mutant alleles of various meiotic genes and HO-mediated DSB formation at a chromosome IV *HO cs* location. Genetic markers and calculation of apparent crossover frequency among the total number of two-spore-viable dyads (n) are as described in Table 2 and Figure 3. A Fisher's Exact test was used to determine whether values differ significantly from the control for the *HO cs5* or the *HO cs7* strains.

Genotype (Strain)	Phenotype of *spo11 spo13* dyads									n	2x apparent CO events	% apparent CO	*P*
	Leu+ | Leu+			Leu+ | Leu−			Leu− | Leu−						
	Thr+ | Thr−	Thr− | Thr−	Thr+ | Thr+	Thr+ | Thr−	Thr− | Thr−	Thr+ | Thr+	Thr+ | Thr−	Thr− | Thr−	Thr+ | Thr+				
*cs5* (LY207)	162	10	1384	0	0	0	0	0	0	1556	324	20.8	**‐**
*cs5 zip1* (LY288)	39	1	331	0	0	0	0	0	0	371	78	21.0	0.9433
*cs5 zip2* (LY289)	36	6	370	0	1	0	0	0	1	414	72	17.4	0.1292
*cs5 zip3* (LY341)	60	10	939	0	2	0	0	0	0	1011	120	11.9	<0.0001
*cs5 msh4* (LY299)	27	1	404	0	1	0	0	0	1	434	54	12.4	<0.0001
*cs5 mlh3* (LY363)	68	1	1029	0	2	0	0	0	0	1100	136	12.4	<0.0001
*cs5 mlh3 zip3* (LY413)	20	2	285	0	0	0	0	0	0	307	40	13.0	0.0016
*cs5 mlh3 msh4* (LY410)	41	1	421	0	0	0	0	0	0	463	82	17.7	0.1468
*cs5 mer3* (LY376)	22	1	282	0	0	0	0	0	0	305	44	14.4	0.0095
*cs5 P_CLB2_-MMS4* (LY291)	27	0	327	0	0	0	0	0	0	354	54	15.3	0.0179
*cs5 yen1 P_CLB2_-MMS4* (LY388)	32	0	281	0	0	0	0	0	0	313	64	20.4	0.9392
*cs5 slx1 mlh3 yen1 P_CLB2_-MMS4* (LY868)	47	0	518	0	0	0	0	0	0	565	94	16.6	0.0357
*cs5 sgs1-ΔC795* (LY850)	93	3	620	0	0	0	0	0	1	717	186	25.9	0.0080
*cs7* (LY324)	46	0	464	0	0	0	0	0	0	510	92	18.0	‐
*cs7 zip3* (LY382)	29	2	388	0	0	0	0	0	0	419	58	13.8	0.0891
*cs7 slx1 mlh3 yen1 P_CLB2_-MMS4* (LY871)	49	12	508	0	0	0	0	0	0	569	98	17.2	0.7492

We note that the observed reduction in crossing over in *zip3*, *msh4*, *mlh3* and *mer3* mutants could reflect a selective diminishment of nonreciprocal recombination events that involve extremely long conversion tract lengths, instead of a diminishment in reciprocal crossovers. Under this scenario, the effect of Zip3, Msh4, Mlh3 and Mer3 meiotic crossover-associated proteins on HO-initiated meiotic recombination intermediates would be to promote and/or stabilize longer conversion tracts. To address this possibility, we used PCR analysis to assess the fraction of apparent crossovers resulting from a reciprocal *vs.* a nonreciprocal event in the *zip3mlh3* double mutant strain, which displays a ∼37% reduction in apparent interhomolog crossovers in our *cs5* experimental strain. We found that 20% (4/20) of apparent interhomolog crossovers in *zip3mlh3* dyads (compared to 29% of apparent crossovers in wild-type) remain the result of long-tract interhomolog conversion events, which possibly arise through a BIR mechanism. This result indicates that the ∼37% reduction in apparent interhomolog crossovers caused by the combined absence of Zip3 and Mlh3 does not reflect a selective diminishment of nonreciprocal recombination events.

Factors that process joint molecule recombination intermediates independent of an SC-associated pathway include Mms4, the Slx1/Slx4 complex, and Yen1 (De Los Santos *et al.* 2003; Fricke and Brill 2003; Ip *et al.* 2008; Jessop and Lichten 2008). To assess if HO DSBs on chromosome IV rely on any of these so-called “class II” recombination factors, we examined HO-mediated crossing over at *HO cs5* in *spo11* strains deficient in meiotic Mms4 and Yen1 (*P_CLB2_-MMS4* and *P_CLB2_-MMS4yen1* double mutants). We found that HO-mediated interhomolog recombination is not significantly diminished in the absence of these proteins (Figure 4; Table 4). Finally, we created a strain in which both SC-associated and SC-independent classes of recombination factors are missing. Together, Mlh3, Yen1, Mms4 and Slx1/Slx4 proteins have been implicated in ensuring the resolution of most Spo11- mediated crossovers during wild-type meiosis (Zakharyevich *et al.* 2012). Simultaneous removal of these four factors did not, however, significantly reduce HO-mediated, apparent crossovers at *cs5*, nor *cs7* in our *spo11spo13* meiotic cells (Figure 4, Table 4).

The Sgs1 helicase has both pro- and anti-crossover activity and its absence allows multi-chromatid exchange events during meiosis (Rockmill *et al.* 2003; Jessop *et al.* 2006; De Muyt *et al.* 2012). Expression of *sgs1-C795*, a meiotic null allele (Mullen *et al.* 2000; Rockmill *et al.* 2003), results in a mild increase in HO-mediated apparent crossover recombination at *HO cs5*, from 20.8 to 25.9% *P* = 0.008; Figure 4, Table 4). Thus, Sgs1 may limit HO-mediated interhomolog recombination in *spo11* meiotic cells.

These data, however, need to be considered in light of the unexpected result that *mlh3msh4* double mutants and our *slx1mlh3yen1 P_CLB2_-MMS4* quadruple mutant exhibit no significant decrease in HO-mediated crossing over, despite the reduction in crossing over observed in *msh4* and *mlh3* single mutants. While this observation could reflect *bona fide* genetic interactions between crossover promoting factors, it may instead indicate that non-specific strain background effects modulate the recombination phenotype.

Altogether, our data suggest that in meiotic cells devoid of Spo11, HO-mediated interhomolog recombination intermediates might engage with a subset, but not the full cohort of SC associated (class I) crossover factors. Furthermore, HO-mediated meiotic DSBs do not absolutely rely on either class I or class II crossover proteins for interhomolog repair.

### An HO-mediated DSB located proximal or distal to the centromere is not sufficient to promote stable pairwise associations between homologous centromeres during meiosis

We targeted HO DSBs to budding yeast’s longest chromosome in order ask whether the position of an interhomolog recombination event with respect to the centromere determines homolog pairing outcomes in meiosis. One homolog pairing process is the recombination-dependent transition from homology-independent centromere “coupling” to homologous centromere pairing (Tsubouchi and Roeder 2005; Stewart and Dawson 2008). We used *lacO*-associated GFP-LacI and *tetO*-associated TetR-mCherry to assess whether HO-mediated recombination, proximal or distal to *CENIV*, promotes homologous centromere pairing in *spo11* mutant meiosis.

**Figure 5:**
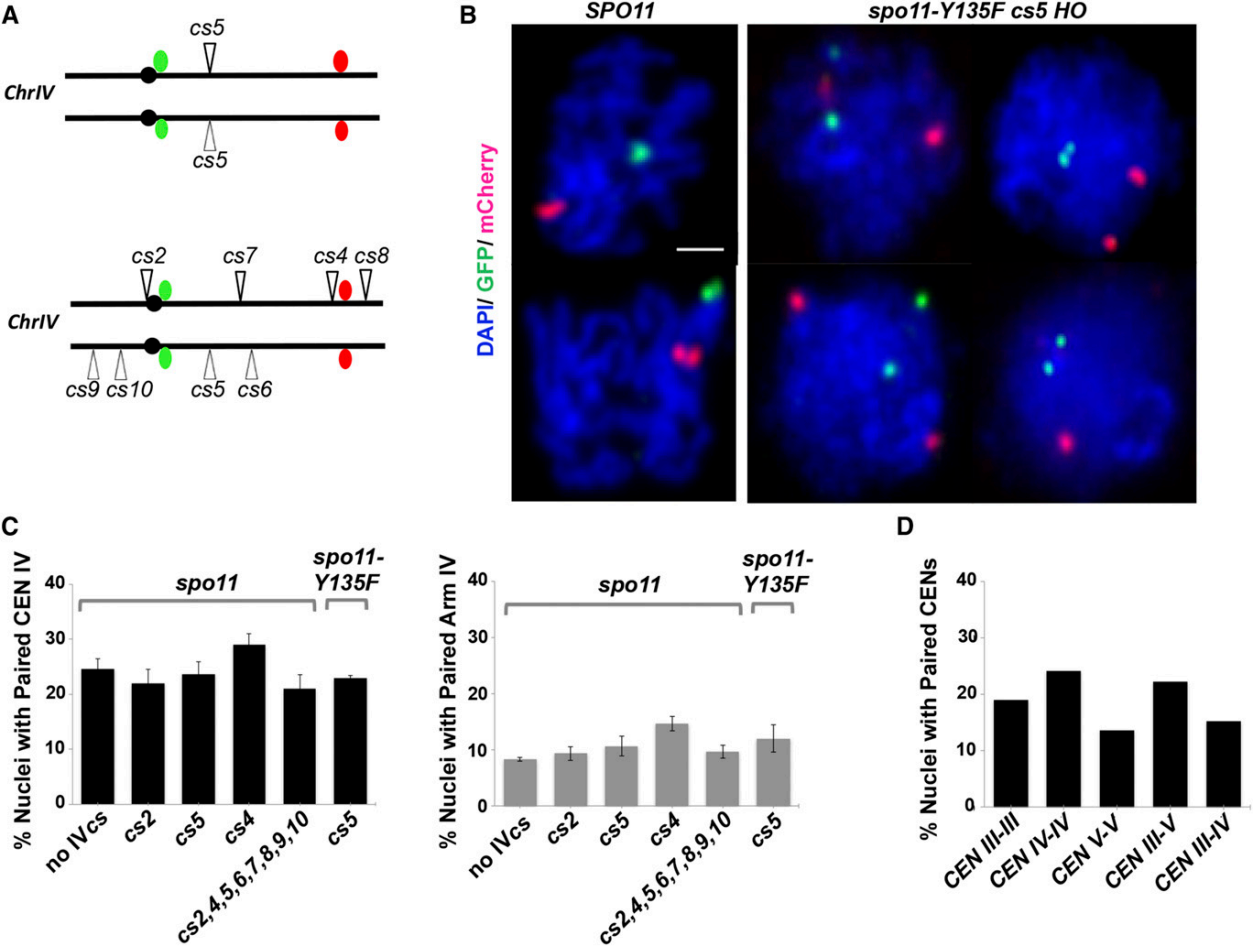
A centromere-proximal or distal HO DSB is not sufficient to pair homologous chromosomes. (A) Cartoons show the locations of *lacO* DNA sequences 705 bp to the right of centromere IV (green) and *tetO* DNA sequences at 1,242 kb on chromosome IV in strains used for cytology experiments. GFP-LacI and TetR-mCherry, expressed in *trans*, bind *lacO* and *tetO* respectively. Strains homozygous for a single *HO cs* (top) or carrying seven active *HO cs* loci (bottom) were utilized in cytological experiments. (B) Images show surface spread meiotic nuclei from wild-type strains (LY42; left column), and from *spo11-Y135F* strains expressing meiosis-specific HO endonuclease and carrying *HO cs5* (LY887; center and right columns) at 15 hr. of sporulation. The presence of Hop1 (not shown) was used to select meiotic nuclei for pairing analysis. The distance between foci corresponding to LacI-GFP bound to *lacO* sequences near *CEN IV* (green), and TetR-mCherry bound to *tetO* sequences on the arm of chromosome IV were considered paired if foci center to foci center < 0.5 μm apart. Bar, 1μm. (C) Bar graphs display the average frequency of *CEN IV* pairing or chromosome IV arm pairing at 15 hr of sporulation in control (LY176) and chromosome IV *HO cs* – carrying strains (left to right: LY176, LY173, LY174, LY175, LY331and LY887). 100 meiotic nuclei per genotype were analyzed in triplicate (n = 300 total per genotype). Bars depict standard error of the mean. (D) Homologous and non-homologous centromere pairing between centromeres indicated on the *x* axis was assessed in *spo11* mutant strains (left to right: LY303, LY176, LY356, LY357 and LY358) at 15 hr of sporulation (n > 100).

*lacO* DNA sequences were integrated 705 nucleotides to the right of *CENIV*, and *tetO* DNA sequences were introduced at coordinate 1,242 kb on chromosome IV (See Materials and Methods). We visualized *lacO* and *tetO* DNA sequences on surface spread meiotic chromosomes by *lacO*- or *tetO*-binding proteins GFP-LacI or TetR-mCherry, expressed in *trans* within the same strains. These cytological tools were built into diploids carrying *HO cs2*, *cs4*, or *cs5*, and into a strain carrying seven active chromosome IV *HO cs* loci (although this strain is also heterozygous for *cs6*, which (presumably) is not actively cut; Figure 5A). Diploid strains were sporulated for ∼15 hr before preparing surface-spread nuclei for immunofluorescence; an antibody that targets the meiosis-specific chromosomal protein Hop1 was utilized to verify that nuclei had progressed into meiosis.

We found that among meiotic nuclei from control *spo11* strains lacking HO endonuclease, 25% exhibited homologous pairing between the centromere regions of chromosome IV (Figure 5B, C). This frequency of association is higher than expected if pairwise centromere associations between 32 individual chromosomes are random and completely independent of homology in *spo11* mutants, thus we also examined pairing between the centromere regions of chromosomes III and V, and between centromeres III, V, and IV, in *spo11* mutant meiotic cells. Consistent with previous studies that found a preferential association between centromeres belonging to chromosomes of similar size (Lefrançois *et al.* 2016), centromeres III and V were paired in 22% of surface-spread nuclei (n >100), while centromeres III and IV (which have a greater size differential) displayed a 15% pairing frequency (n > 100; Figure 5D).

Having established the baseline of centromere IV pairing in *spo11* meiotic cells, we next asked whether strains with HO-mediated DSBs on chromosome IV display an increase in homologous centromere pairing. We found that *spo11* mutants carrying a single *HO cs* in conjunction with HO endonuclease exhibited a similar frequency of paired centromeres IV as *spo11* cells devoid of HO endonuclease, even when the *HO cs* is adjacent to the *CENIV* locus. Homologous centromeres IV were paired in 22%, 29% and 24% of meiotic cells at 15 hr of sporulation from strains carrying *cs2*, cs*4*, or cs*5*, respectively (n= 300; Figure 5B, C). Furthermore, homologous centromeres IV were paired in 21% of meiotic cells from strains carrying seven active chromosome IV *HO cs* loci (*cs2*, *cs4*, *cs5*, *cs7*, *cs8*, *cs9* and *cs10*; n= 300; Figure 5B, C).

Similarly, pairing at a chromosome IV arm location, detected in about 8% of *spo11* control cells devoid of HO endonuclease, was detected at approximately the same frequency in *spo11* cells carrying single or multiple *HO cs* loci and meiotic HO endonuclease: Chromosome IV arm pairing was observed in 9%, 15%, and 11% of meiotic cells from *HO*-expressing strains carrying *cs2*, *cs4* and *cs5*, respectively, and in 10% of the meiotic cells from a strain carrying seven active chromosome IV *HO cs* loci (Figure 5B, C).

We conclude that a single HO-mediated recombination event, while capable of promoting interhomolog recombination in ∼20% of meioses, is not sufficient to promote stable pairwise associations between homologous centromeres, even when positioned 250 nucleotides from the centromere.

### An HO-mediated meiotic DSB fails to promote SC assembly in the context of a null or catalytically inactive spo11 allele, even when meiotic axis proteins are overexpressed

Initial steps in meiotic recombination are required for the assembly of SC in budding yeast, which, at early time points, occurs predominantly from centromere regions (Tsubouchi *et al.* 2008). An earlier study reported that HO-mediated recombination between homologous chromosomes III was unaccompanied by SC assembly in *spo11* meiotic cells (Malkova *et al.* 2000), but the small size of chromosome III leaves open the possibility that a partial SC assembly event was missed in this prior experiment. We thus asked whether SC structure(s) assemble on the largest yeast chromosome (IV) in response to an HO-mediated DSB, and furthermore whether the distance from the centromere of the HO-mediated recombination event matters.

**Figure 6:**
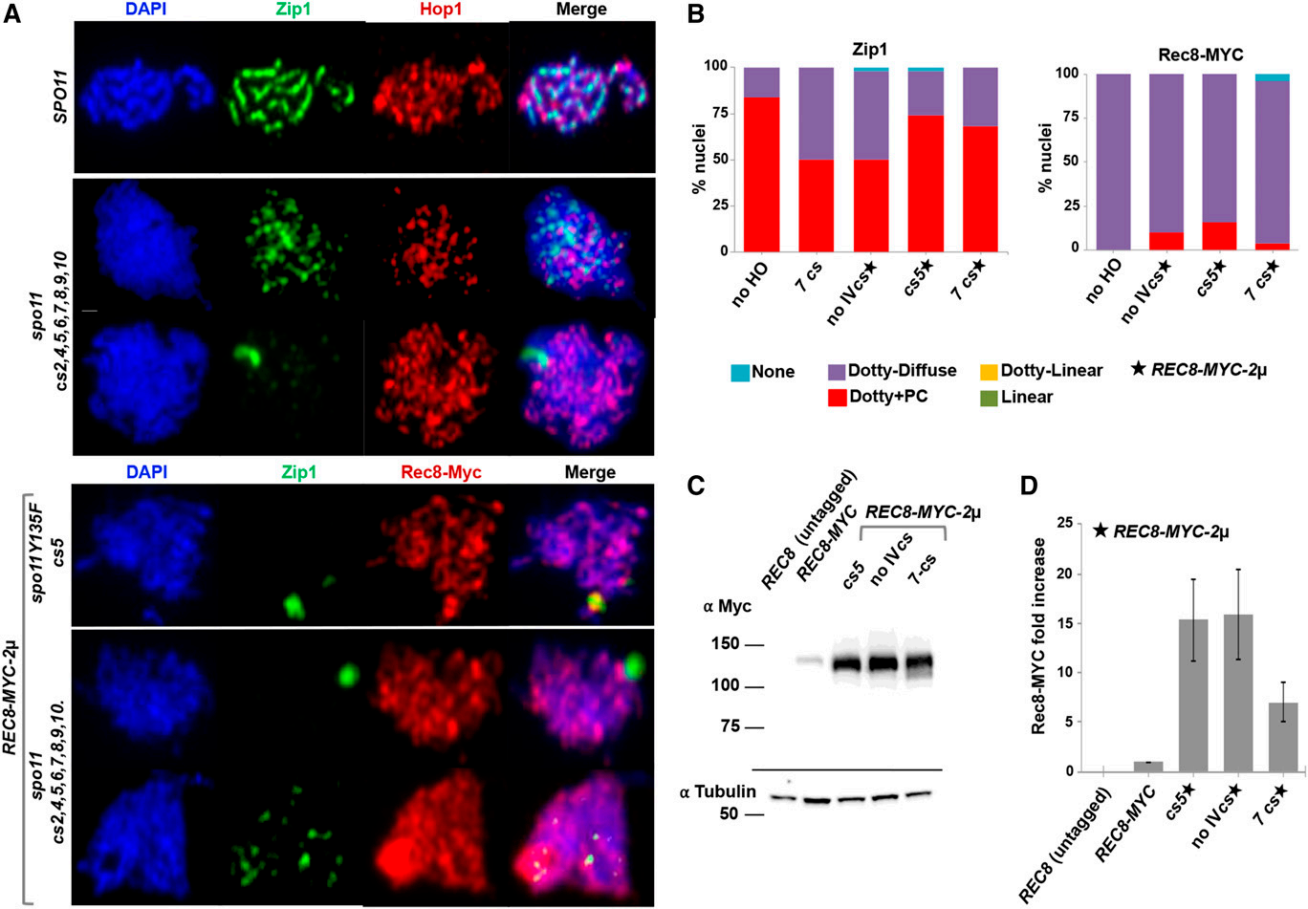
Synaptonemal complex does not assemble in response to an HO-mediated meiotic DSB. (A) Representative surface spread meiotic nuclei from *SPO11* (top row; YAM424), or *spo11* null strains carrying seven active *HO cs* loci (*cs2*, *cs4*, *cs5*, *cs6*, *cs7*, *cs8*, *cs9*, *cs10*; LY371) at 15 hr of sporulation (upper panel, second and third row). Lower panel displays a representative nucleus from the *spo11-Y135F* strain carrying *HO cs5* and overexpressing Rec8 (LY890; lower panel, top row) and two representative nuclei from the *spo11* null strain carrying seven active *HO cs* and overexpressing Rec8 (LY892, lower panel, bottom two rows) at 24 hr of sporulation in *ndt80* strains. Zip1 (green) binds diffusely to and also assembles some bright foci on DAPI-stained meiotic chromatin (blue) from these *spo11* strains, regardless of HO-induced meiotic DSBs; polycomplex aggregates of Zip1 (white arrowheads) are often observed. The presence of the meiosis-specific Hop1 protein or Rec8-MYC (red) is displayed in the third column. Bar, 1μm. (B) The proportions of nuclei (n = 50) with different Zip1 and Rec8-MYC distribution phenotypes at 24 hr of sporulation are plotted for a control strain missing *P_SPO13_-HO* (LY893), the *spo11* null strain carrying seven active *HO cs* loci (LY371), a Rec8-overexpression control *spo11-Y135F* strain with no chromosome IV *HO cs* (LY891), the *spo11-Y135F* strain carrying *HO cs5* and overexpressing Rec8 (LY890) and the *spo11* null strain carrying seven active *HO cs* loci and overexpressing Rec8 (LY892). Immunoblot in (C) shows Rec8-MYC levels in meiotic cells at 24 hr of sporulation from a control *SPO11 ndt80* strain in which *REC8* is untagged and which happens to also be homozygous for *P_GAL_-HOP1* (LY769), a control *spo11-Y135F ndt80* strain homozygous for *REC8-MYC* (LY893), followed by *REC8*-overexpressing LY890, LY891 and LY892 strains. The same cultures were used to prepare meiotic surface spread nuclei analyzed at the 24 hr time point. Molecular weight indicators are given (kDa) to the left. (D) Graph plots Rec8-MYC protein levels from a strain carrying two endogenous copies of *REC8-MYC* and strains carrying 2µ-*REC8-MYC*. Tubulin levels were used to normalize Rec8-MYC levels across samples. The average of 3 replicates is plotted; bars give standard error of the mean.

Surface-spread meiotic nuclei from HO-expressing and control *spo11* strains at 15 hr of sporulation were labeled with antibodies against the SC transverse filament protein Zip1 to visualize SC, and with antibodies against the meiosis-specific chromosomal protein Hop1 to verify that nuclei had progressed into meiosis (Figure 6A). In *spo11* meiotic nuclei, Zip1 localizes diffusely on surface-spread chromosomes, with several brighter foci likely corresponding to centromeres (Tsubouchi and Roeder 2005). A large fraction of *spo11* control nuclei also display an aggregate of Zip1 called a polycomplex. Zip1’s distribution pattern on meiotic chromatin from *spo11* strains expressing *HO* and carrying seven active *HO cs* loci (including *cs2*, positioned immediately adjacent to *CENIV*) appeared indistinguishable from *spo11* control meiotic nuclei (Figure 6A, B). The absence of linear Zip1 structures on meiotic chromatin indicates that HO-mediated recombination is incapable of interfacing with and/or successfully activating the molecular pathway(s) that facilitate SC assembly in *spo11* meiotic nuclei.

Spo11 requires 9 additional accessory proteins to initiate recombination during meiosis (Lam and Keeney 2014). A catalytically inactive mutant version of Spo11, Spo11-Y135F, is capable of localizing to chromatin during meiosis (Prieler *et al.* 2005). With the hope of targeting Spo11 partners to an HO-mediated DSB, we initially created a gene fusion between Spo11-Y135F and the N terminus of HO endonuclease in our *HO cs*-containing *spo11* cells. However, the chimeric protein did not exhibit HO endonuclease activity, as indicated by the absence of meiotic interhomolog recombination at the *MAT* locus in strains carrying this fusion. We next analyzed strains sustaining HO-mediated, meiotic DSBs with *spo11-Y135F* expressed *in trans*. We found no evidence of homologous centromere pairing nor SC assembly in meiotic nuclei carrying meiotic HO-mediated DSBs at *cs5*, nor in strains carrying seven active *cs* loci and homozygous for *spo11-Y135F* (Figures 5, 6A), indicating that the Spo11-Y135F protein cannot confer to HO-mediated DSBs the capacity to promote meiotic chromosome pairing processes.

SC assembly requires the meiosis-specific cohesin, Rec8 (Klein *et al.* 1999). It was previously reported that artificial DSBs, applied to *spo11* mutant meiotic cells that overexpress Rec8, lead to Zip1 linear assemblies which were interpreted to be tripartite SC (Brar *et al.* 2009). This result led us to wonder whether the overexpression of a meiosis-specific cohesin or another chromosome axis protein would enable HO-mediated DSBs to promote SC assembly. To address this question, we introduced a 2-micrometer plasmid carrying *REC8-MYC* driven by the *HOP1* promoter in order to overexpress *REC8-MYC* in *HO*-expressing *spo11* meiotic cells carrying no *HO cs*, a single *HO cs*, or seven active *HO cs* loci. A western blot demonstrated that Rec8-MYC is approximately five times more abundant in our *REC8-MYC* overexpression strains relative to the level produced by two chromosomal copies of *REC8-MYC* (Figure 6C). However, Zip1’s distribution on surface-spread chromosomes from these meiotic cells appeared dotty-diffuse regardless of whether the strain expressed higher than wild-type levels of Rec8-MYC (Figure 6A, B). Thus, the level of Rec8-MYC overexpression achieved in this experiment fails to bestow HO-mediated DSBs with the capacity to promote SC assembly.

We also asked whether the overexpression of the meiosis-specific chromosome axis proteins Hop1 and Red1 results in HO-mediated SC assembly in *spo11* meiotic nuclei. A western blot confirmed that Hop1 is overexpressed at least ninefold relative to control cells in *spo11* cells carrying a *HOP1-RED1* 2-micrometer plasmid (a kind gift of N. Hollingsworth; (Hollingsworth and Ponte 1997) (Figure S1). Immunofluorescence on surface-spread nuclei indicated that, with or without HO-mediated DSBs, *spo11* cells carrying the *HOP1-RED1* 2-micrometer plasmid exhibited a dotty-diffuse Zip1 distribution on chromatin and/or a Zip1 polycomplex, with no linear SC-like structures (Figure S1).

### Artificial DSBs supplied en masse by exposure to phleomycin influence SC protein distribution but fail to promote robust SC assembly in spo11 meiotic nuclei

We wondered whether the failure of our meiotic HO-mediated DSBs to support centromere pairing or SC assembly is due to the fact that these processes need a minimal threshold level of DSBs in order to progress. In order to address this question, we examined pairing and SC protein distribution in meiotic nuclei from *spo11* mutants that had sustained multiple DSBs due to phleomycin exposure. Whereas surface-spread meiotic nuclei from *spo11* mutants rarely exhibit more than one or two Rad51 foci, a significant increase in Rad51 foci (ranging between 5-55; *P* ≤ 0.001; Figure S2A) was observed upon exposing *spo11* meiotic nuclei to an increasing series of phleomycin doses. The dozens of Rad51 foci observed reflect a snapshot of one stage in the repair of phleomycin-dependent DSBs, and are likely an underestimate of DSBs formed in this experiment. We observed a statistically insignificant increase in homologous pairing at centromeres and arm regions of chromosome IV in strains that experienced the highest dose of phleomycin (*P* = 0.378; Figure S2B). The vast majority of meiotic nuclei exposed to high doses of phleomycin display a “dotty-diffuse” distribution of SC structural proteins Zip1 and Ecm11 (similar to that seen in the negative control), although a small fraction (∼10%) of phleomycin-exposed nuclei display multiple short linear assemblies of coincident Zip1 and Ecm11 (Figure S2C, D). However, nuclei from cells that had been exposed to high doses of phleomycin only rarely display the Zip1 polycomplex structure that is frequently observed in control nuclei. Taken together, these results indicate that exposure of *spo11* meiotic cells to phleomycin causes a change in the distribution of Zip1 protein such that Zip1 is less likely to form a polycomplex structure, and may weakly facilitate homologous associations between chromosomes and minimal SC-like assemblies, but does not trigger robust homologous synapsis.

## Discussion

The current study was motivated by an interest in the molecular mechanism that connects meiotic recombination to processes that generate and reinforce homolog pairing in budding yeast. The HO endonuclease was found to promote interhomolog crossover recombination in *spo11* yeast meiosis at higher frequency than in mitotic cells (Malkova *et al.* 1996b; Malkova *et al.* 2000), consistent with the idea that Spo11-independent features of the meiotic nucleus may allow DSBs to engage with chromosome pairing and/or recombination pathways that promote favorable outcomes for meiosis; an example of such a mechanism might be the interhomolog bias-promoting activity of meiosis-specific chromosome axis proteins like Red1 and Rec8, and/or meiosis-specific recombinase machinery (Schwacha and Kleckner 1997; Hong *et al.* 2013). However, the presence of Spo11 has also been found to exert a positive effect on the capacity of artificially-supplied meiotic DSBs to repair as interhomolog crossovers (Malkova *et al.* 1996b; Malkova *et al.* 2000; Neale *et al.* 2002; Medhi *et al.* 2016). The extent to which Spo11 activity is uniquely engaged with and capable of driving meiotic chromosome pairing processes remains obscure.

Our investigation into how meiotic DSB repair is coordinated with chromosome pairing in meiotic cells speaks to several questions: Does the position of a DSB relative to the centromere affect the success of early homologous pairing events? Does a threshold level of DSBs need to be met in order to ensure homolog alignment pairing or SC assembly? Are programmed meiotic DSBs specialized in their capacity to promote homolog pairing events? In this study, we asked whether HO-mediated DSBs, many positioned at chromosome IV locations that are frequently cut by Spo11, are capable of facilitating any of the meiotic chromosome pairing processes that normally accompany programmed DSBs. We furthermore asked the same question of DSBs supplied *en masse* by exposure to phleomycin.

### HO-mediated meiotic DSBs on chromosome IV may lack robust repair template bias

Successful homolog segregation in meiosis relies on recombination-based associations between non-sister chromatids; it follows that meiotic recombination is strongly biased toward utilizing the homolog as repair template (Schwacha and Kleckner 1994; Schwacha and Kleckner 1997; Hunter and Kleckner 2001; Hong *et al.* 2013). By contrast, homologous recombination mechanisms in mitotic cells almost exclusively use the sister chromatid (Kadyk and Hartwell 1992). One question that our experiments highlight is to what extent might a Spo11-associated DSB be specialized in its capacity to preferentially engage the homolog?

One can explore this question by asking whether HO-mediated, meiotic DSBs exhibit a meiotic-like or mitotic-like preference in repair template choice. An earlier study demonstrated that HO DSBs frequently access the non-sister chromatid for repair (Malkova *et al.* 2000), and our observation of interhomolog repair of meiotic HO DSBs on chromosome IV is consistent with the idea that HO DSB repair in meiotic cells has a more “meiotic” *vs.* “mitotic” repair template bias.

However, considering the frequency of HO-mediated DSB formation in our system, we suggest that HO-mediated DSB repair in *spo11* meiotic cells exhibit a modest to strong inter-sister repair template bias. Southern blot analysis of total HO-mediated DSBs in our system suggests that 20–60% of total DNA is cleaved in strains homozygous for a given *HO* cut site; this suggests that, on average, one or two (out of four) chromatids are cut by HO in every meiosis. Presuming a random resolution process that does not favor a crossover outcome, the detected ∼15–20% HO-mediated interhomolog crossover events per meiosis suggests that ∼30–40% of meioses may have involved an HO meiotic DSB that repaired off of the homolog (half resulting in a crossover, and half resulting in a non-crossover). In fact, the aforementioned study that analyzed HO-mediated meiotic DSB repair determined that, in the absence of Spo11, interhomolog repair may be biased toward a non-crossover outcome: In this earlier study which measured both crossover and noncrossover interhomolog DSB repair events, only 27% of total HO–mediated interhomolog recombination events gave a crossover outcome in the absence of Spo11 (*vs.* 52% in the presence of Spo11) (Malkova *et al.* 2000). Taking this into consideration, perhaps as high as ∼50% of meioses involved interhomolog repair of an HO break in our system. As mentioned in the Results, our Southern blots may underestimate the total number of HO-mediated DSBs generated, due to the possibility of hyperresected DSBs in the *rad51* background. However, even if we assume one HO-mediated DNA break per meiotic cell in our *spo11* populations, these estimations suggest that HO-mediated meiotic DSBs utilize the sister chromatid with a higher probability than would be expected in the absence of bias (no bias corresponds to an interhomolog:intersister repair ratio of 2:1).

Moreover, since HO-mediated meiotic interhomolog recombination remains robust in *red1*, *dmc1*, and *mek1* mutants, the interhomolog to intersister repair template bias we infer for HO-mediated DSBs in *spo11* meiosis is likely to be similar to the repair template bias associated with Spo11-mediated DSBs in the absence of Red1, Dmc1 or Mek1 proteins, which has been reported to be strongly biased toward the sister chromatid (Schwacha and Kleckner 1997; Hong *et al.* 2013).

Our results are consistent with the idea that, relative to the HO DSBs examined in our system, at least a subset of Spo11-mediated DNA breaks are more successful at preferentially engaging the homolog for repair.

### Distinct roles for MRX complex components in the repair of a non-Spo11 meiotic DSB

The Mre11, Rad50, and Xrs2 proteins have been implicated in assembling a complex (MRX) which facilitates the formation and repair of Spo11-mediated DSBs (Alani *et al.* 1990; Johzuka and Ogawa 1995; Tsubouchi and Ogawa 1998; Bressan *et al.* 1999). Separation-of-function *mre11* and *rad50* alleles indicate that these factors have evolved distinct activities that are independently critical for Spo11 DSB formation and repair (Alani *et al.* 1990; Tsubouchi and Ogawa 1998); on the other hand, bypass of *xrs2* null meiotic phenotypes by artificial localization of Mre11 to the nucleus suggests that Xrs2 is indirectly involved in Spo11 DSB formation and repair through its singular capacity to recruit Mre11 (Oh *et al.* 2016).

One conclusion we draw from our analysis of *mre11* and *xrs2* mutants is that MRX components are differentially relied upon for the repair of at least a subset of HO-mediated DSBs during meiosis. The HO-associated spore inviability phenotype observed in *spo11spo13mre11* is not observed in *spo11spo13xrs2* strains, suggesting that a subset of HO-mediated meiotic DSBs critically depend upon Mre11 but not Xrs2 for their repair. In light of the idea that Xrs2’s role in MRX function during meiosis is thought to be through its capacity to recruit Mre11 to the nucleus, the successful repair of HO-mediated meiotic DSBs in *spo11spo13xrs2* meiocytes implies that Xrs2 is not required to recruit Mre11 to the HO-mediated DNA damage that relies on Mre11 for repair.

Second, we observed robust meiotic interhomolog recombination at *MAT* in both *spo11spo13mre11* and *spo11spo13xrs2* strains expressing *P_SPO13_-HO*. This result suggests that while a subset of HO-mediated meiotic DSBs critically depends upon Mre11 for their repair, neither Mre11 nor Xrs2 is essential *per se* for the interhomolog repair of HO-mediated meiotic DSBs. A prior study’s finding that Xrs2 and Rad50 are dispensable for HO-mediated meiotic DSB repair at *MAT* (Malkova *et al.* 1996b) is consistent with our observation and furthermore indicates that each MRX component is dispensable for the interhomolog repair of at least some HO-mediated meiotic DSBs when Spo11 is absent. This is in contrast to Spo11-mediated meiotic DSBs, which rely on Mre11 and Rad50, and possibly Xrs2, for their repair during meiosis (Alani *et al.* 1990; Nairz and Klein 1997; Tsubouchi and Ogawa 1998; Borde 2007; Gobbini *et al.* 2016).

Our data also indicates that while dispensable for repair *per se*, Xrs2 nevertheless influences the repair outcome of meiotic HO DSBs, at least those formed at *HO cs5*. Apparent crossover levels on chromosome IV diminished by 74% at *HO cs5* in *spo11spo13* mutants devoid of Xrs2. The fact that HO-mediated interhomolog conversions at the *MAT* locus remained unchanged in the *xrs2* mutant relative to the control (*XRS2*) strain leads us to suggest that Xrs2 activity may engage with HO-mediated interhomolog recombination intermediates in a manner that promotes a crossover outcome. However, we note that data from earlier studies, while not completely conclusive, indicates little effect of Rad50 or Xrs2 on the likelihood of a crossover outcome associated with HO-mediated meiotic recombination at the *MAT* locus (if one compares HO-mediated crossover outcomes at the *MAT* locus determined for *rad50* mutants and inferred for *xrs2* mutants (Malkova *et al.* 1996b), with crossover outcomes at the *LEU2* locus determined for *spo11* mutants (Malkova *et al.* 2000)). Taking these earlier results into consideration, diminished HO-mediated crossing over at *cs5* among diploid dyads from *spo11spo13xrs2* strains may not be due to Xrs2’s involvement in the crossover/noncrossover decision, but instead may reflect a role for Xrs2 in promoting interhomolog (over intersister) repair at certain select DSB locations in the genome, for example at *cs5* but not at *MAT*.

### HO-mediated meiotic DSBs may be accessed by a subset of class I recombination proteins

Malkova *et al.* (2000) demonstrated that HO-mediated meiotic interhomolog repair events in *SPO11+* cells rely on the meiosis-specific crossover promoting complex, MutSγ, to a similar extent as Spo11-mediated meiotic DSBs. Interestingly, this prior study also provided evidence that Spo11 activity can confer on HO DSBs, *in trans*, an increased capacity to resolve as a crossover. Medhi *et al.* (2016) also reported evidence in support of the idea that Spo11 increases the likelihood that an artificially-supplied meiotic DSB will repair via a “meiotic-like” crossover pathway. However, whether artificial DSBs utilize meiosis-specific crossover factors, such as MutSγ, when Spo11 is absent was not examined in prior studies. We find that Msh4-Msh5 (MutSγ), as well as Zip3, Mlh3 and Mer3 may influence a substantial fraction (∼40%) of HO-mediated meiotic interhomolog repair events even when Spo11 is absent, but Zip1 and Zip2 have little or no influence over the interhomolog repair of HO-mediated, meiotic DSBs. Taken together, our results and the results of Malkova *et al.* (2000) suggest that additional factors, apart from access of the HO-recombination intermediate to MutSγ *per se* must be involved in Spo11’s capacity to promote a crossover outcome to interhomolog HO DSB repair events in meiotic cells; we speculate that one of these factors could be proper engagement with a class I recombination complex that includes the Zip1 and Zip2 (and associated) proteins.

### Artificial DSBs are a poor substitute for yeast Spo11 in promoting homologous pairing or synapsis from centromeres

We are particularly interested in the molecular features of a meiotic DSB that facilitate the process of homologous centromere pairing. In budding yeast, centromeres may be the first regions of meiotic chromosomes to undergo stable homologous alignment in response to recombination, since SC assembly initiates earliest from centromere regions (Tsubouchi *et al.* 2008). However, centromere pairing is also independent of SC assembly as it remains intact in mutants that are missing certain building block components of the SC, such as Ecm11 and Gmc2 (Kurdzo *et al.* 2017). Our data indicate that artificial DSBs, generated close to or far from a centromere, and either delivered as singular events by the HO endonuclease or *en masse* by the radiomimetic drug phleomycin, are unable to promote stable homologous centromere pairing in *spo11* meiotic cells. This data suggests that (at least a subset of) Spo11-associated recombination events are uniquely specialized to facilitate this chromosome reorganization process.

Synapsis normally initiates from centromere regions as well as from interhomolog recombination events along chromosome arms (Chua and Roeder 1998; Agarwal and Roeder 2000; Henderson *et al.* 2004; Tsubouchi *et al.* 2008). A prior study reported that meiotic HO DSBs on the small chromosome III fail to promote SC assembly (Malkova *et al.* 2000). Similarly, despite their capacity to promote interhomolog recombination, we observed that HO DSBs positioned at Spo11 hotspot locations close to or far from the centromere in *spo11* meiotic cells are incapable of even partial SC assembly on the long chromosome IV.

Our data thus reveal that HO-mediated DSBs, while capable of promoting interhomolog crossover recombination during meiotic prophase, are nevertheless *incapable* of promoting homologous centromere pairing or robust SC assembly. These observations indicate that the artificial DSBs examined in this study are processed in a fundamentally different manner from at least a subset of programmed (Spo11-mediated) DSBs.

It is important to bear in mind that not all Spo11-mediated DSBs are necessarily capable of promoting homologous centromere pairing and synapsis during wild-type meiosis; some programmed meiotic DSBs may be processed similarly to the artificially-supplied DSBs examined in this study. This consideration does not diminish the importance of understanding the molecular basis that underlies the difference between programmed and artificially-supplied meiotic DSBs with respect to their capacity to drive homologous pairing and synapsis.

An alternative explanation for the failure of artificially-supplied DSBs to drive meiotic chromosomal outcomes, which does not involve a specialized function of Spo11, is that either the chromosomal position, timing, or overall abundance of DSBs is the critical feature that determines whether one or more DSBs can promote homologous centromere pairing and/or SC assembly. Indeed, the extent of synapsis in meiotic nuclei has been observed to be directly correlated with Spo11 DSB abundance (Henderson *et al.* 2004; Brar *et al.* 2009; Rockmill *et al.* 2013); this correlation could be explained by a reliance of homologous synapsis mechanisms on the chromosomal position, timing, or background abundance of one or more programmed DSBs.

While it is formally possible that either chromosomal position, timing or overall DSB abundance is the primary reason for the failure of artificially-supplied DSBs to promote centromere pairing and synapsis in *spo11* meiotic nuclei, this explanation alone is challenging to reconcile with our experimental data. First, in stark contrast to even *spo11* hypomorphic mutants that have severe reductions in programmed DSB abundance (Henderson *et al.* 2004; Brar *et al.* 2009; Rockmill *et al.* 2013), the HO-mediated DSBs in our experiments uniformly fail to promote even partial SC assembly outcomes. This is true even in the context of prolonged arrest provided by an *ndt80* null mutation; the removal of the Ndt80 transcription factor prevents progression beyond mid-late meiotic prophase and theoretically would allow HO-mediated DSBs to accumulate and give incipient SC assembly events additional time to develop into mature SC stretches, as is reflected by the *ndt80*-mediated rescue of synapsis in *spo11* severe hypomorphs (Rockmill *et al.* 2013). Second, our high-dose phleomycin experiments likely generate dozens to hundreds of DSBs in meiotic nuclei that are at multiple stages of prophase (as our strains progress through meiosis asynchronously), but phleomycin-induced DSBs fail to promote robust homologous centromere pairing or synapsis in any *spo11ndt80* meiotic nuclei examined after a prolonged duration (24 hr) in sporulation conditions. Thus, while not unequivocally ruling out alternative models, our experimental data favors the possibility that Spo11-association may be a critical prerequisite for a meiotic DSB to engage with the molecular mechanisms that drive centromere pairing and SC assembly.

### A possible molecular basis for the capacity of meiotic DSBs to promote homolog pairing processes

How might pro-centromere pairing/synapsis DSBs differ from meiotic DSBs that fail to promote pairing and synapsis outcomes? Homologous centromere pairing may be regulated by the phosphorylation of Zip1 by the Mec1 kinase. Prior to meiotic recombination initiation in budding yeast meiosis, Zip1 mediates the pairwise association of centromeres without regard to homology (Tsubouchi and Roeder 2005). Falk *et al.* (2010) demonstrated that Zip1’s capacity to mediate centromere association is abolished by phosphorylation of serine residue 75; these authors propose that Mec1-mediated phosphorylation of Zip1 might serve to promote the dissolution of centromere coupling events in coordination with recombination initiation, facilitating the subsequent formation of homologous centromere interactions. The failure of HO- or phleomycin-induced meiotic DSBs to promote robust homologous centromere pairing may not be due to the absence of Mec1 kinase activity *per se*, however, as artificial DSBs, like Spo11 DSBs, have been reported to activate Mec1 and Tel1 kinases (Lydall *et al.* 1996; Usui *et al.* 2001; Cartagena-Lirola *et al.* 2006) and to phosphorylate the meiosis-specific kinase, Mek1 during meiosis (Wan *et al.* 2004; Cartagena-Lirola *et al.* 2008). Instead, the presence of activated Mec1 in a local, specialized chromosomal context might serve to influence centromere behavior. Alternatively, or in addition, perhaps successful pro-centromere pairing DSB events are specialized in activating a second regulatory factor required in addition to activated Mec1 for the dissolution of non-homologous centromere associations, or for the re-establishment of centromere pairing between homologs.

While our experiments do not reveal a mechanism, our data do offer some clues that may be mechanistically relevant for understanding how certain meiotic DSBs are uniquely capable of facilitating synapsis. SC assembly normally occurs downstream of a discrete intermediate step in the homologous recombination process (Henderson *et al.* 2004; Zickler and Kleckner 2015). As discussed above, certain meiosis-specific recombination factors (such as Zip3, Mer3, Msh4 and Mlh3) appear to functionally influence HO-mediated, meiotic recombination intermediates in the absence of Spo11. However, Zip3, Mer3, Msh4 and Mlh3 are unlikely to be acting on HO recombination intermediates in their normal “class I” manner since other factors in the same recombination pathway, such as Zip1 and Zip2, do not similarly engage with HO-mediated recombination intermediates, at least when Spo11 is absent. Importantly, while Zip1, Zip2, Zip3 and Msh4-Msh5 act in the same (class I) crossover recombination pathway during wild-type meiosis, Zip1 and Zip2 play a more critical role in SC assembly than Zip3 and Msh4 during wild-type meiosis. Thus, we suggest that the success of a meiotic DSB to promote SC assembly depends upon its early engagement with recombination complexes that properly coordinate Zip3 and Msh4 with the pro-crossover proteins that are essential for SC assembly (Zip1, Zip2, Zip4, Spo16). The engagement of a meiotic DSB with this sort of “complete” class I recombination pathway could dually ensure that 1) the ensuing recombination intermediate is channeled into a stable crossover-associated configuration, and 2) global alignment of homologous chromosomes, via the promotion of processes like centromere pairing and assembly of tripartite SC, is achieved.

Several pieces of evidence also suggest the possibility that pro-synapsis recombination intermediates may uniquely interface with the chromosome axis. While DNA that is most frequently cleaved by Spo11 lies in chromosome “loop” regions, away from axis locations where the meiosis-specific Hop1 and Red1 proteins are enriched (Gerton *et al.* 2000; Blat *et al.* 2002; Blitzblau *et al.* 2007; Pan *et al.* 2011), the axis localization of several of Spo11’s accessory proteins (Panizza *et al.* 2011) and the reliance of meiotic DSB formation on Hop1 and Red1 (Mao-Draayer *et al.* 1996; Schwacha and Kleckner 1997; Xu *et al.* 1997) suggests that many Spo11 DSB sites are physically close to chromosome axes during break formation and subsequent repair steps (Panizza *et al.* 2011). The axis-association of DNA sequences undergoing a DSB is furthermore consistent with the localization of “recombination nodules” (structures associated with ongoing DNA repair visualized by electron microscopy) between intimately-aligned homologous chromosome axes (Zickler and Kleckner 1999). Finally, *hop1* or *rec8* separation-of-function alleles that confer proficiency in meiotic DSB formation are nevertheless defective in homologous synapsis (Klein *et al.* 1999; Carballo *et al.* 2008; Brar *et al.* 2009).

The possibility that axis proximity is a critical prerequisite for a newly-initiated DSB to engage with meiotic-like repair pathways is supported by a recent finding that repair of a VDE-mediated DNA break during meiosis utilizes the meiosis-specific MutLγ complex more frequently when the break is positioned within axis-protein enriched DNA (Medhi *et al.* 2016). Importantly, this effect was found to be dependent on the presence of Spo11 in *trans*. Furthermore, in their classic study, Thorne and Byers noted a role for the meiotic chromosome axis-associated protein, Hop1, in enabling artificial DSBs to rescue the chromosome segregation functions of Spo11 in meiotic cells (Thorne Lw 1993).

Our finding that interhomolog repair of HO-mediated meiotic DSBs in *spo11* meiocytes both fails to promote centromere pairing and SC assembly, and occurs independently of the chromosome-axis associated protein Red1 and the Mek1 kinase, further strengthens the correlation between the chromosome axis and a mechanism that coordinates DSB repair with homologous synapsis in budding yeast. A prior study indicated that artificial DSBs can rescue Spo11’s SC assembly function if the Rec8 cohesin protein is overexpressed (Brar *et al.* 2009), but we were unable to observe robust synapsis by HO-mediated DSBs when Rec8, Hop1 or Red1 proteins were overexpressed in our strain background. While alternative models remain possible, our results together with previously published data support the possibility that Spo11 maintains a specialized capacity to ensure (even *in trans*) that at least a subset of DSBs are processed in the context of axis protein-regulated structures.

### Programmed meiotic DSBs may trigger global chromosome dynamics through a specialized interface with Dmc1-associated factors

Our experiments also reveal the possibility that a constraint to repair using the Dmc1 recombinase may be a unique property of (at least a large subset of) Spo11 DSBs. Interestingly, Smith and colleagues also found that the repair of a set of non-canonical DSBs in fission yeast meiosis also occurs independently of Dmc1, but is dependent on Rad51, unlike programmed meiotic DSBs in fission yeast meiocytes (Farah *et al.* 2005).

Schwacha and Kleckner (1997) characterized Red1 and Dmc1 as important core features of an “interhomolog only” pathway for DSB repair, in which axis-associated Red1 is responsible for coupling the maturation of recombination intermediates to the Dmc1 recombinase. In the absence of Red1, remnant meiotic DSBs do not engage with this “interhomolog only” pathway and instead undergo repair without interhomolog template bias. Similarly, we show that the interhomolog repair of an HO DSB during meiosis does not rely on Red1 nor Dmc1, nor Mek1 (which targets multiple molecular pathways that coordinately promote the use of the Dmc1 recombinase by Spo11-associated meiotic DSBs) and appears to exhibit low interhomolog repair template bias. We therefore speculate that pro-synapsis, Spo11-associated DSBs may differ from HO-associated DSBs in their capacity to productively interface with the Red1-Hop1-Mek1-Dmc1 “interhomolog only” pathway for DSB repair. Artificially supplied DSBs in *spo11* meiotic cells have been shown to induce Mek1 phosphorylation (Cartagena-Lirola *et al.* 2008), indicating that events apart from Mek1 activation are required for artificially-supplied DSBs to properly engage with the “interhomolog only” mechanism.

While we propose that pro-synapsis DSBs may differ from artificially-supplied DSBs in the manner by which they engage with a Dmc1-associated pathway for meiotic recombination, the capacity to promote SC assembly is not likely to be due solely to utilization of the Dmc1 recombinase *per se*. As is particularly evident in strains that progress through meiotic prophase more slowly (such as those of the BR background), the absence of Dmc1 or Mek1 diminishes but does not abolish synapsis, indicating the existence of redundant molecular mechanisms to couple Spo11 DSBs to SC assembly (Rockmill and Roeder 1991; Rockmill *et al.* 1995; Tsubouchi and Roeder 2003). Our *rad51* separation-of-function experiments furthermore indicate that HO-mediated meiotic DSBs are not prohibited from utilizing the Dmc1 recombinase, and we found that forcing phleomycin-induced or HO-induced meiotic DNA breaks to repair using the Dmc1 recombinase (through use of the *rad51-II3A* allele) does not confer a robust capacity to promote synapsis (Figure S3). We speculate that pro-synapsis DSBs may possess the unique capacity to drive homologous centromere pairing and synapsis processes because of a specialized engagement with the Red1/Hop1/Mek1 pathway, but that this capability is at least partly reliant on additional downstream pathway outcomes that are independent of Dmc1.
